# Home Is Where the Hearth Is: Anthracological and Microstratigraphic Analyses of Pleistocene and Holocene Combustion Features, Riwi Cave (Kimberley, Western Australia)

**DOI:** 10.1007/s10816-017-9354-y

**Published:** 2017-10-26

**Authors:** Rose Whitau, Dorcas Vannieuwenhuyse, Emilie Dotte-Sarout, Jane Balme, Sue O’Connor

**Affiliations:** 1 Archaeology and Natural History, School of Culture History and Language, College of Asia and the Pacific, Australian National University, Canberra, Australia; 2 Archaeology M257, University of Western Australia, 35 Stirling Highway, Crawley, WA 6009 Australia; 3 School of Archaeology and Anthropology, College of Social Sciences, Australian National University, Canberra, Australia

**Keywords:** Hearths, Combustion features, Anthracology, Micromorphology, Fuel wood management, Australian archaeology

## Abstract

The manipulation of fire is a technological act. The identification of the archaeological signatures of the controlled use of fire has important implications not only for the estimations of the origins and functions of the first fireplaces but also for our understanding of prehistoric technological development and resource use. At Riwi (Kimberley region, Western Australia), excavations over two field seasons have revealed a discontinuous occupation sequence over the past 45 ka, showing numerous, different combustion features interspersed within the deposit. Anthracological and micromorphological investigations at Riwi Cave indicate that the combustion features at the site can be categorised into three types: flat combustion features (type A), dug combustion features (type B) and thick accumulations of mixed combustion residues (type C). These provide evidence for two kinds of combustion practice: (i) fires lit directly on the ground and most likely not re-used and (ii) ground ovens, the latter appearing some 10,000 years after the first evidence for occupation of the site. A comparison of the wood species identified within these combustion features with those from equivalent scattered context levels, enables an exploration of the potential factors influencing wood selection and fire use through time at the site. A detailed understanding of the relationship between wood charcoal remains and archaeological context yields significant information on changes to environmental context and site occupation patterns over time.

**Electronic supplementary material:**

The online version of this article (10.1007/s10816-017-9354-y) contains supplementary material, which is available to authorized users.

## Introduction

An essential component of the hunter-gatherer tool-kit, fire is a source of light, warmth, protection and an instrument for cooking, manufacturing equipment and altering the environment. The origins and functions of the first fireplaces have important implications for hominin evolution and form a key debate in Palaeolithic archaeology (*e.g.* Alperson-Afil and Goren-Inbar 2010; de Lumley 2006; Goudsblom 1986; Gowlett 2006; Gowlett and Wrangham 2013; James *et al.* 1989; Roebroeks and Villa 2011; Sandgathe *et al.* 2011; Wrangham 2009), with the first habitual, controlled uses of fire linked to increases in brain size and cognition (Brain 1981; Gowlett 2006; Pruetz and LaDuke 2010; Rolland 2004; Wrangham 2009) and the colonisation of the northern latitudes (Brace *et al.* 1987; Gowlett 2006; Oakley 1956; Preece *et al.* 2006; Rolland 2004; Stratus 1989; Weiner *et al.* 1998; Wrangham *et al.* 1999). Evidence for anthropogenic fire can be contextually variable and, in the case of the earliest examples, highly contentious (*e.g.* Berna *et al.* 2012; for a recent review on the evidence of human use and control of fire, see Goldberg *et al.* 2017; Stahlschmidt *et al.* 2015, pp. 181–183). The most unambiguous signature for the habitual, controlled use of fire is the structured hearth, with the earliest evidence found in Qesem Cave in Israel, dated approximately to 400 ka (Karkanas *et al.* 2007; Shahack-Gross *et al.* 2014).

The identification of the archaeological signatures of hearth-building processes has important implications not only for the estimation of the first controlled uses of fire but also for understanding of prehistoric technological development and resource use. A suite of techniques for both the macro- and micro- scale analyses of combustion structures are currently employed, ranging from the *in situ* description of hearth structures (*e.g.* Metcalfe and Heath 1990; Solé *et al.* 2013; Vaquero and Pastó 2001), to the physical and chemical analysis of charred components and sediments with the application of geophysical (*e.g.* Barbetti 1986; Bellomo 1991, 1993), geochemical (*e.g.* Karkanas *et al.* 2002; Rudner and Sumegi 2002), micromorphological (*e.g.* Mallol *et al.* 2013a; Mentzer 2014; Schiegl *et al.* 2004; Wattez 1992; *cf.* review in Aldeias 2017) and anthracological analyses (*e.g.* Beauclair *et al.* 2009; Henry and Théry-Parisot 2014; Scheel-Ybert *et al.* 2014; Vidal-Matutano 2016). The study presented in this paper combines the latter two approaches, anthracology and micromorphology, to explore building processes of hearths from an Australian Indigenous archaeological context.

Microstratigraphic investigations of combustion features can help document the anthropogenic activities associated with their formation and also assess their degree of preservation or alteration (Mallol *et al.* 2013a; Mentzer 2014; Wattez 1992), by identifying their components (Estévez *et al.* 2014; March *et al.* 2014; Mentzer 2014; Stiner *et al.* 1995; Wattez 1988; Weiner *et al.* 1995), deciphering whether they are intact or disturbed (Goldberg *et al.* 2009; Mallol *et al.* 2013a; Mentzer 2014; Miller and Sievers 2012; Miller *et al.* 2010), documenting how they have affected the substrate (Aldeias *et al.* 2016; Canti and Linford 2000; Mallol *et al.* 2013b) and to what extent they have been affected by post-depositional processes (Karkanas 2010; Karkanas *et al.* 2000; Karkanas *et al.* 2002; Mentzer 2014). Soil morphology experiments and ethnoarchaeological investigations have identified various characteristics of combustion features (Courty *et al.* 1989; Macphail *et al.* 2004; Mallol *et al.* 2007; Wattez 1992; Miller *et al.* 2013) that have been applied to archaeological contexts. These include distinguishing single from multiphase hearth use (Meignen *et al.* 1989, 2007), discriminating hearths from secondary ash dumps (Schiegl *et al.* 2003) and detecting burned stable layers (Macphail *et al.* 2004).

Anthracological investigations of wood charcoal assemblages follow the premise that hearth features and concentrations of dense charcoal are episodic archaeological contexts. They are the primary refuse of the last few firing events, whereas dispersed wood charcoal from scattered contexts are secondary refuse, potentially accrued over a more protracted period of time (Asouti and Austin 2005; Byrne *et al.* 2013; Chabal 1990, 1992; Chabal *et al.* 1999; Dotte-Sarout *et al.* 2015; Théry-Parisot *et al.* 2010). Where wood charcoal analyses are employed for palaeoenvironmental reconstruction, hearths and concentrated charcoal features must be avoided, and scattered charcoal from occupation contexts must be examined in order to represent the accumulation of multiple fuel wood collection events, so that more of the site’s surrounding environment will be represented (Badal-García *et al.* 2012; Chabal *et al.* 1999; Dufraisse 2012, 2014; Théry-Parisot *et al.* 2010; Scheel-Ybert 2002). Concentrated features, such as charcoal lenses and hearths, should be analysed in conjunction with scattered contexts to tease out the cultural factors that influence the creation of a charcoal assemblage at a site (Asouti and Austin 2005; Byrne *et al.* 2013; Théry-Parisot *et al.* 2010). However, in spite of this premise which is essential to the discipline, very few anthracological studies have explicitly employed micro-scale techniques to define the combustion context of a site in order to understand combustion processes better (*e.g.* Allué *et al.* 2017; Damblon *et al.* 1996; Damblon and Haesaerts 2002; Vidal-Matutano 2016).

**Fig. 1:**
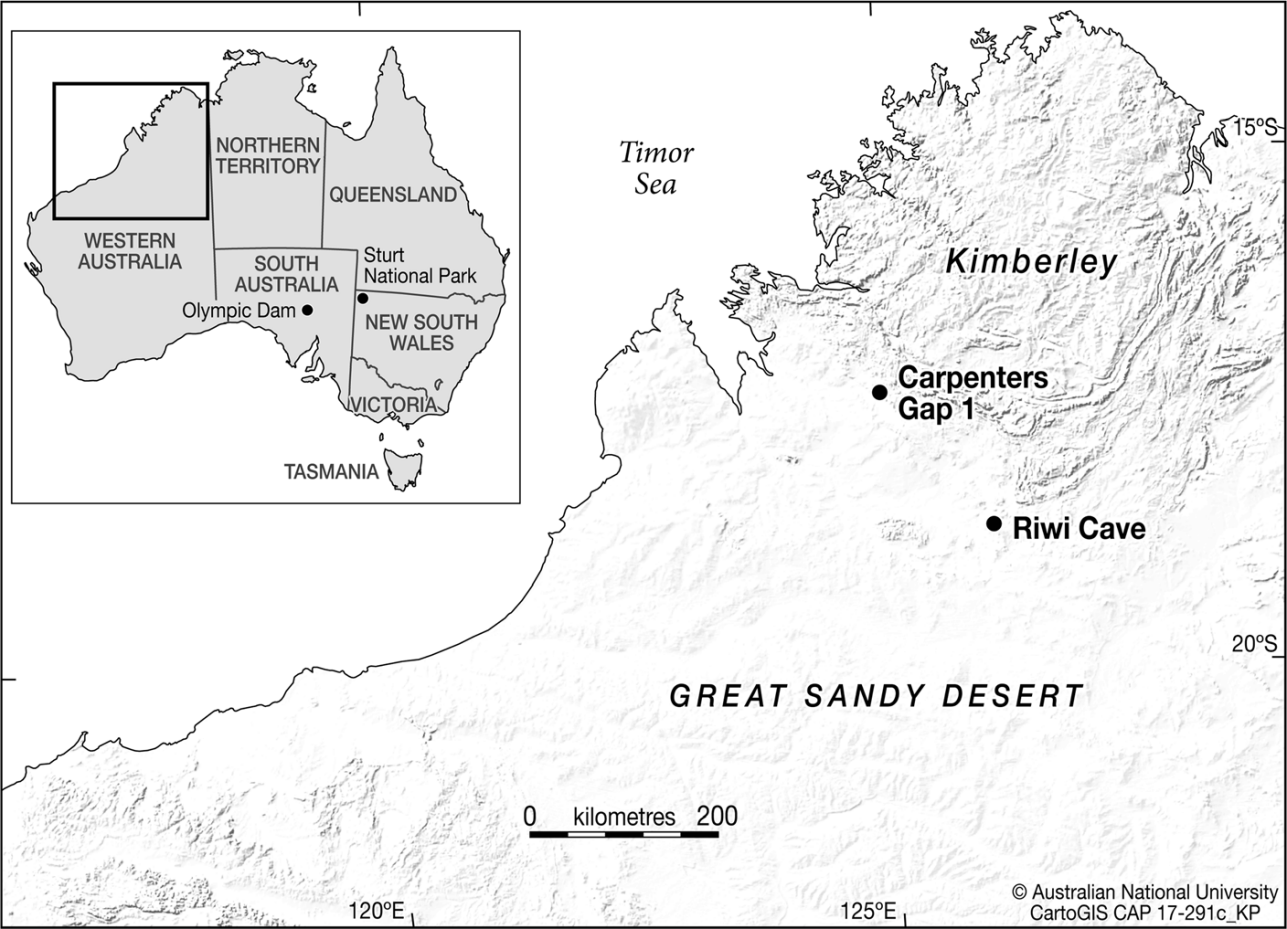
Northern Western Australia with inset of Australia and sites mentioned in the text (CAD: CartoGIS, Australian National University)

In the Australian archaeological context, various geoarchaeological and archaeological analyses have explored the formation and post-depositional alterations of hearth features at open sites, particularly at Sturt National Park in arid western New South Wales (Fig. 1; Fanning and Holdaway 2001; Fanning *et al.* 2008, 2009; Holdaway *et al.* 2017) and Olympic Dam in northeastern South Australia (Fig. 1; Sullivan *et al.* 2012; Sullivan and Hughes 2013). Analysis of sedimentary processes within Australian rock shelters has concentrated largely on preservation potential (Ward 2004; Ward and Larcombe 2003; Ward *et al.* 2006) and the vertical movement of artefacts (Allen and O’Connell 2003; Bird *et al.* 2002; Hiscock 1985, 1990). With the exception of micromorphological analyses conducted at Carpenters Gap 1 rockshelter (Fig. 1; Vannieuwenhuyse 2016; Vannieuwenhuyse *et al.* 2017), which revealed the presence of combustion features’ rake-out zones in the Late Pleistocene and Holocene archaeological levels, few attempts have been made in Australian archaeology to understand how fireplaces were built, maintained and used by Indigenous hunter-gatherer populations. Similarly, very few anthracological investigations have been conducted in Australian contexts, although the number of statistically viable analyses is starting to improve (see Byrne *et al.* 2013; Carah 2010; Dotte-Sarout *et al.* 2015; King 2015; Whitau *et al.* 2016a).

In this paper, we present the results from combined anthracological and micromorphological analyses of combustion features at Riwi Cave in the southern Kimberley region of northern Western Australia. At Riwi, excavations have revealed a discontinuous occupation sequence over the past 45 ka showing numerous different combustion features interspersed within the deposit (Balme 2000; Vannieuwenhuyse 2016; Whitau *et al.* 2016a; Wood *et al.* 2016). This sequence represents an exceptional opportunity to undertake a combined and detailed geo-anthracological analysis in order to explore the depositional and post-depositional factors that have affected the creation and preservation of hearths. We propose a typology of these features based on their sedimentological and anthracological characteristics. The wood species identified within the hearths are compared with the spectrum of charcoal identified from contemporaneous scattered contexts, which are more representative of the broad vegetation changes in the vicinity of the site (see anthracological analysis in Whitau *et al.* 2016a). The combined anthracological and micromorphological approaches enable an investigation of the possible factors influencing wood selection, fire-use and deep-time combustion features related practices in an Australian archaeological context.

### Site Description, Context and Antiquity

Riwi Cave is located in the southern Kimberley region of Western Australia (Fig. 1), within the traditional lands of the Indigenous Gooniyandi people, on the northern edge of the Great Sandy Desert. Located in the South Lawford Range and formed within Devonian Pillara and Sadler limestone facies (Playford *et al.* 2009, p. 251), the cave is situated within a valley enclosed by low-range outcrops, through which an ephemeral creek flows during the wet season. Receiving a sub-tropical to semi-arid climate within the 500-mm isohyet of the Australian Summer Monsoon (Bureau of Meteorology 2015), the skeletal soils of the Riwi valley support a low tree steppe: hummock grassland (*Triodia bitextura*) with scattered bloodwood (*Corymbia dicromophloia/Corymbia opaca*) and snappy gum (*Eucalyptus brevifolia*). A variety of dry rainforest associated taxa, including *Celtis strychnoides*, *Dodonaea polyzyga* and *Flueggea virosa* grow along the limestone range and outliers (Whitau *et al.* 2016a). The cave is composed of two chambers, and a channel running through the north-west side of the front chamber, where a yellow ball flower tree (*Mallotus nesophilus*) grows, signifies water circulation in the cave during the wet season (for site review and plan, see Vannieuwenhuyse 2016; Wood *et al.* 2016; Whitau *et al.* 2016a). Vestigial evidence of past human occupation includes rock art on the cave walls and lithic artefacts scattered across the floor of the cave’s entrance.

**Fig. 2:**
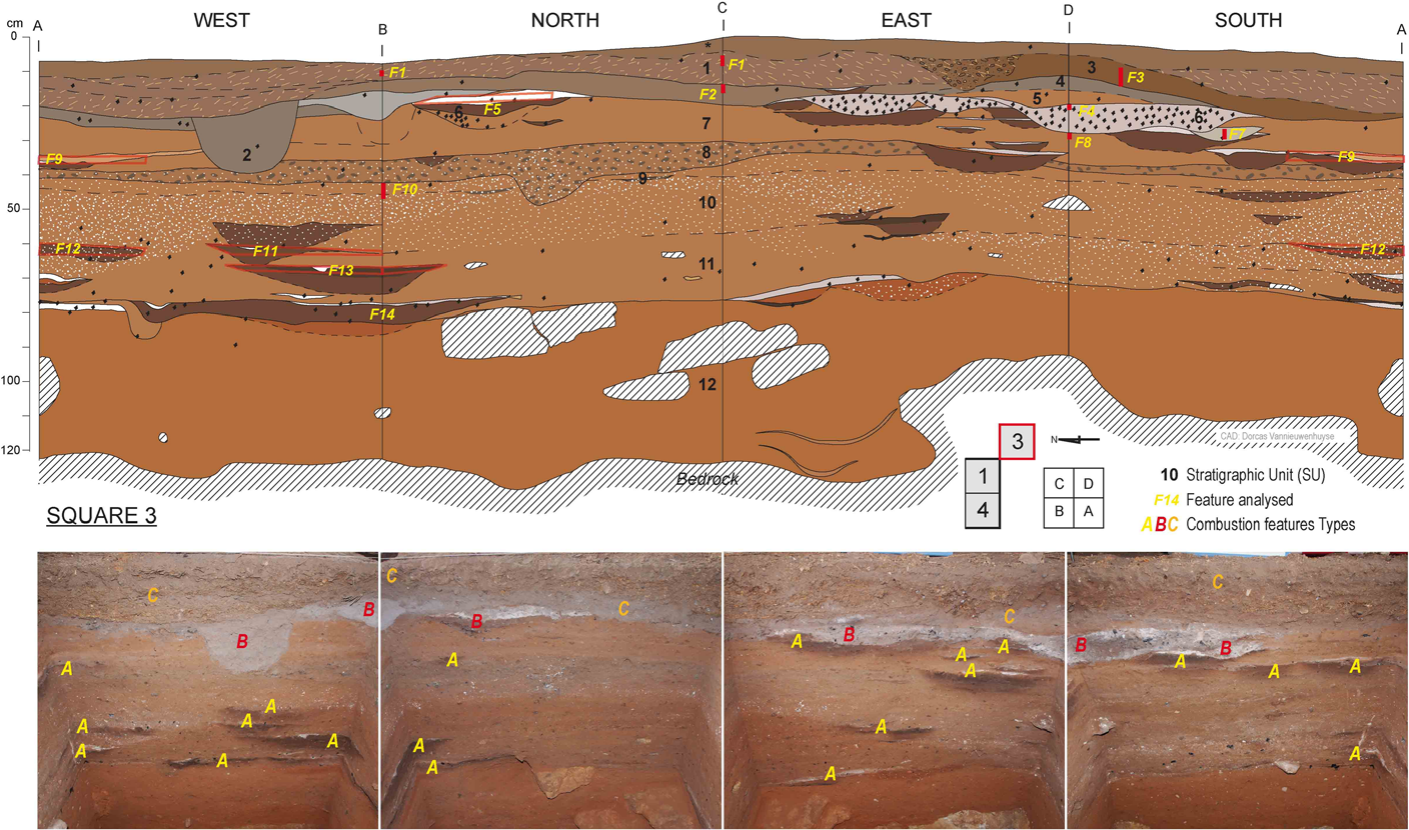
Riwi square 3 section and photos showing the provenience of combustion features and scattered charcoal assemblages used for the anthracological analysis. Refer to text and Fig. 4 for combustion features typology (photos and CAD: Dorcas Vannieuwenhuyse)

**Fig. 3:**
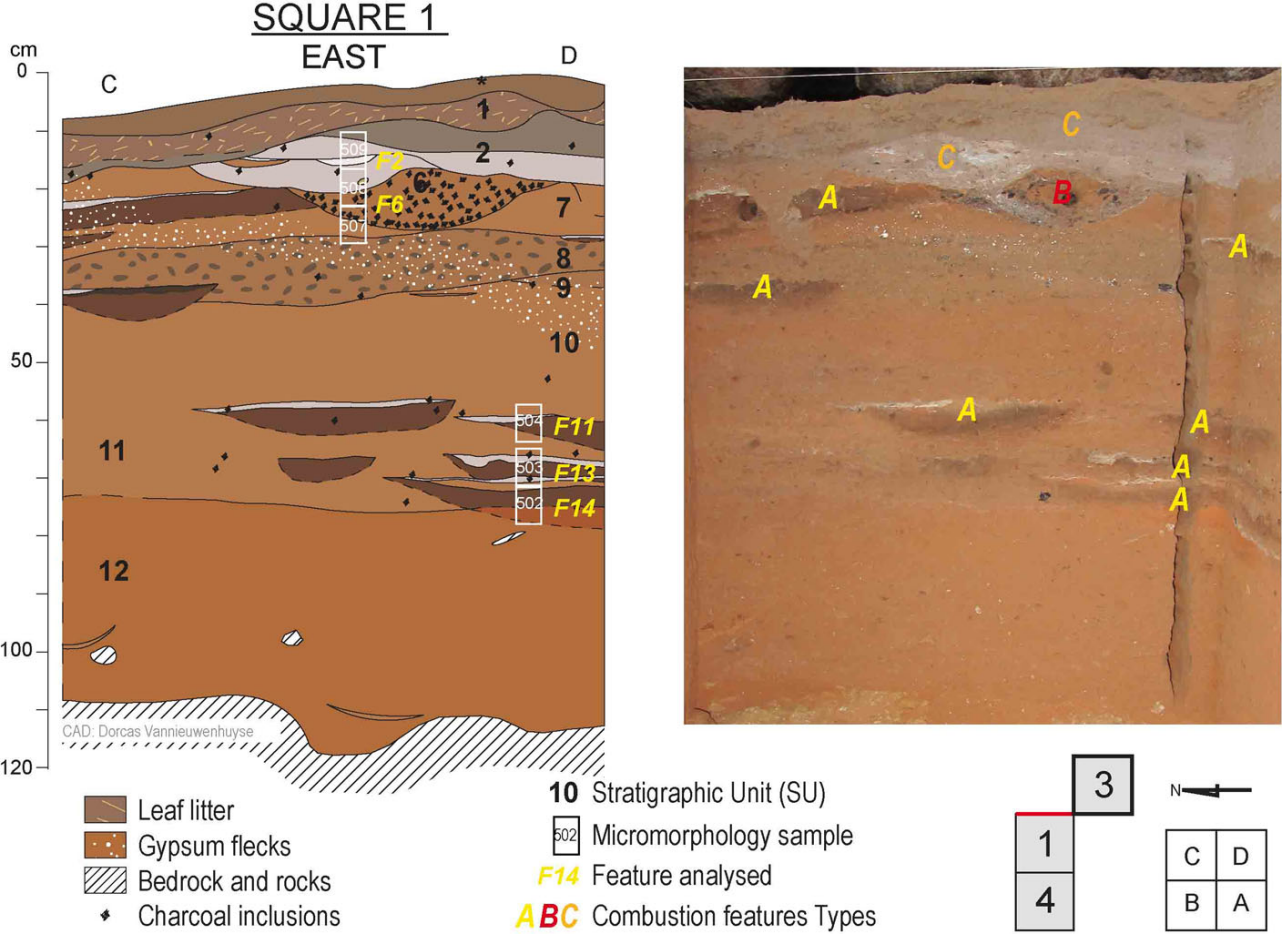
Riwi square 1 sections and photos showing combustion features sampled for the micromorphological analysis. Refer to text and Fig. 4 for combustion features typology (photos and CAD: Dorcas Vannieuwenhuyse)

Riwi Cave was first excavated in 1999 (Balme 2000). In 2013, the original 1 m^2^ test pit (square 1) was emptied and the excavation area expanded, with the addition of 2 × 1 m^2^ test pits inside the cave (squares 3 and 4) and one 1 m^2^ test pit at the entrance of the cave (square 5). All squares were taken to bedrock in 50 cm quadrants in arbitrary 2 cm units, reaching an approximate maximum depth of 115 cm in squares 1, 3 and 4 (Figs. 2 and 3). With the exception of certain features, which were removed separately, and bulk sediment samples, which were collected for each excavation unit, all excavated materials were sieved though nested 5 and 1.5 mm screens. Because the Holocene units were desiccated, flotation was avoided. A detailed description of both the cave and the excavation specifics can be found in Whitau *et al.* (2016a) and Wood *et al.* (2016).

A precise radiocarbon chronology, coupled with a detailed optically stimulated luminescence (OSL) chronology, provides one of the most accurately dated archaeological sequences in Australia (Wood *et al.* 2016). The two chronologies are largely consistent throughout the sequence, both identify earliest occupation of the site around 46.4–44.6 ka cal BP (95.4% probability range) at the top of SU12, and both confirm the presence of several chrono-stratigraphic hiatuses in the upper levels of the sequence (Vannieuwenhuyse 2016; Wood *et al.* 2016). The radiocarbon chronology was mainly based on charcoal sampled from the numerous combustion features interspersed within the Riwi archaeological levels (Figs. 2 and 3). Detailed information about the provenance and stratigraphic context of the dated charcoal was gathered by the geoarchaeological observations undertaken in the field. The anthracological analysis provided background information on charcoal wood species and the context of charcoal production (see Table 1). These data are usually limited or not available in the construction of radiocarbon chronologies in Australian archaeological contexts (Ward *et al.* 2016), which are often only based on the age of charcoal fragments with little reference to the context of that charcoal’s production (Wood 2015; Wood *et al.* 2016). The limited reporting of these data is in spite of recent work conducted elsewhere that characterises the spatial relationship between charcoal and charred seeds selected for radiocarbon analysis and associated archaeological materials and features (Asscher *et al.* 2015; Boaretto 2015; Boaretto *et al.* 2009; Rebollo *et al.* 2011; Toffolo *et al.* 2012).

**Table 1:** Combustion features from Riwi squares 1, 3 and 4 analysed for the study

Features—details							Radiocarbon dates associated				
Feature	Feature description	Type	SU	SQ	XU/Quad	Microm sample	Lab. code	Sampling context	Wood species	Radiocarbon age	Modelled calibrated age range 2σ
F1-C	Brown grey layer (mix of very fine sand with abundant leaf litter, charcoal and ash)	C	1	3	2B, 3C		SANU-43337	From sieve	*Grevillea/Hakea* sp.	670 ± 20	655–555
F2-C	Grey-brown ashy layer with charcoal and few leaves	C	2	3	6C		SANU-39505	From sieve	*Corymbia* sp.	6385 ± 30	N/D
				1	D	*R509; R508 top*	D-AMS004061	From feature (wall)	Not determined	6250 ± 35	7245–7010
F3-A	Grey to black fine to very fine ash with abundant charcoal inclusions	A	3	3	4D, 5D		S-ANU38226	From feature (excavation)	*Corymbia* sp.	16,930 ± 50	20,620–20,040
							SANU-38814	From feature (excavation)	*Corymbia* sp.	16,850 ± 100	20,620–20,040
F4-B	Compact white ash with abundant charcoal inclusions in concave pit	B	6	3	6B		S-ANU35920	From feature (wall)	*Corymbia* sp.	30,110 ± 200	34,380–33,720
							D-AMS 004070	From feature (wall)	Not determined	30,154 ± 141	34,370–33,780
F5-B	Hearth with packed chunks of charcoal in large concave pit, interspaces filled by ashes, very sharp boundary with SU7	B	6	3	8D		SANU-39506	From feature (wall)	*Corymbia* sp.	29,050 ± 180	34,040–33,170
F6-B	Hearth with packed chunks of charcoal in large concave pit, interspaces filled by ashes, very sharp boundary with SU7	B	6	1	D	*R508 bottom, R507 top*	S-ANU35907	From feature (wall)	*Corymbia* sp.	29,790 ± 190	34,180–33,580
F7-B	Compact ash with a high density of burnt bone and charcoal in the southeast corner	B	7	3	10D						Between end of SU7 (36,040–34,130) and start of SU6 (34,920–33,850)
F8-A	Ashy sediment with charcoal inclusions, diffuse boundary with burnt red sediment	A	7	3	11D						Between end of SU7 (36,040–34,130) and start of SU6 (34,920–33,850)
F9-A	Small hearth with burnt sand, ash and abundant charcoal	A	7	3	13A						Between end of SU8 (38,130–36,010 and start of SU7 (37,670–35,590 37,934–35,807)
F10-A	Black, compact, fine sediment, with gypsum nodules (2–5 mm)	A	9/10	3	16B, 17B		S-ANU35919	From feature (wall)	Unidentifiable	34,000 ± 310	38,850–37,740
F11-A	Compact dark sediment with charcoal inclusions	A	10/11	3	23B						Between end of SU11 (45,080–42,110) and start of SU10 (44,060–40,490)
				1		*R504*					
F12-A	Well-defined black, compact sediment with charcoal inclusions and gypsum nodules (2–5 mm)	A	10/11	3	23A						Between end of SU11 (41,692–38,671) and start of SU10 (40,018–37,932)
F13-A	Well-defined black, compact sediment with charcoal inclusions and gypsum nodules (2–5 mm)	A	11	3	25B						Within SU11, between 45,420–43,900 and 45,080–42,110
				1		*R503*	ANUA-13005	From level (excavation)	Not determined	41,300 ± 1020	45,180–43,410
F14-A		A	11/12	1		*R502*	SANU-35909	From feature (wall)	*Corymbia* sp. (type R01)	41,590 ± 760	45,900–44,470

A short description is given for each feature with associated combustion features types, stratigraphic context (SU) and related excavation units (XU) from where anthracological (A) and micromorphological (M) samples were collected. When available, radiocarbon dates directly associated with the feature or same stratigraphic level are given. Radiocarbon dates are mentioned calibrated against SHCal13 (Hogg *et al.* 2013) in OxCal v.4.2 (Bronk Ramsey, 2009) following radiocarbon chronology done for the site by Wood *et al.* (2016)

## Materials and Methods

### Sampling

The Riwi anthracological and micromorphological samples were collected from the July 2013 excavations and from the archaeological material recovered and sorted in the laboratory. Twelve combustion features from Riwi square 3 were selected for anthracological analysis (Fig. 2) in conjunction with the analysis of scattered contexts from squares 3 and 4 presented in Whitau *et al.* (2016a). Six micromorphological samples targeting combustion features were extracted from the eastern sections of square 1 (Fig. 3). The proximity of the sampling and the similarities in type of combustion features observed between the two squares allows the comparison and incorporation of both anthracological and micromorphological results to build our analysis. Table 1 presents an overview of the different combustion features and scattered charcoal assemblages analysed for the study, their stratigraphic and sampling context, their typology and their antiquity. Radiocarbon ages follow those of Wood *et al.* (2016) and are given as modelled calibrated values calculated using OxCal 4.2, using SHCal13 or Marine13 calibration curves (Bronk Ramsey 2013; Ramsey and Lee 2013; Ramsey *et al.* 2013; Reimer *et al.* 2013).

### Microstratigraphic Methods

Oriented micromorphological sediment samples were extracted using plaster bandages (Goldberg and Macphail 2003, 2005), which were then prepared following the standard fabrication processes for soil thin sections (Camuti and McGuire 1999; Courty *et al.* 1989; FitzPatrick 1984). Resin impregnation of these monoliths was undertaken at the geotechnical facilities of the School of Earth and Environment at the University of Western Australia. Small pre-cut chips (54 × 63 × 10 mm) were sent to Spectrum Petrographics (Vancouver, Washington, USA) for thin sectioning. Thin sections were digitally scanned for archival and publication purposes (Arpin *et al.* 2002; De Keyser 1999). Thin section observations and microphotography were carried out using a Nikon polarising petrographic microscope in the School of Earth and Environment at the University of Western Australia. Observations were made under different magnifications (×10, ×25, ×50, ×100, ×500) using both plane polarised (PPL) and cross polarised light (XPL). Descriptions follow the terminology standardised by Stoops *et al.* (2010). Identification and interpretation of components and pedofeatures are based on the available micromorphology literature (primarily Bullock *et al.* 1985; Courty *et al.* 1989; Goldberg and Macphail 2005; Stoops *et al.* 2010) and case studies as cited in the text.

### Anthracological Methods

All anthracological analysis was conducted at the Department of Archaeology and Natural History at the Australian National University. Charcoal was identified by snapping fragments along the transverse, radial and tangential longitudinal sections, with the aid of a scalpel where necessary (following Leney and Casteel 1975). An Olympus BH-2 reflected lightfield/darkfield microscope was used to examine exposed sections at magnifications of ×20–500. Rare types and archetypal examples of taxa were selected for further observation and imaging with a JEOL JCM-6000 Neoscope scanning electron microscope (SEM). Following Chabal (1990, 1992) and Théry-Parisot *et al.* (2010), quantification was conducted by count, rather than weight. All of the charcoal fragments over 2 mm were examined from each feature (see feature list in Table 1), except F1-C and F2-C, which are Riwi’s two Holocene stratigraphic units (SU1 and SU2 in Whitau *et al.* 2016a). F1-C and F2-C are composed of an accumulation of combustion features mixed with other natural inputs, wherein features could not be sampled separately due to their thin morphology and the palimpsest nature of their deposition. All of the excavated sediment from F1-C and F2-C was collected during excavation within squares 3 and 4, with all of the > 1.5 mm charcoal fragments transported to the laboratory for anthracological analysis. A minimum of 300 identifiable fragments was sampled for the Holocene units, and a riffle-box was used to split the samples to ensure an unbiased coverage of size differentiation.

Following Whitau *et al.* (2016a), charcoal fragments are described as indeterminate when type-level identification cannot be positively assigned and indeterminable where fragments cannot be positively identified due to degradation. Archaeological material was compared with the reference material housed at the Australian National University described by Whitau *et al.* (2016a). Wood identification keys including Hope (1998), Ilic (1991) and two online databases: Inside Wood (http://insidewood.lib.ncsu.edu/) and the University of Queensland Online Archaeology Collections (http://uqarchaeologyreference.metadata.net/archaeobotany/list) were also employed to aid identification.

## Results

**Fig. 4:**
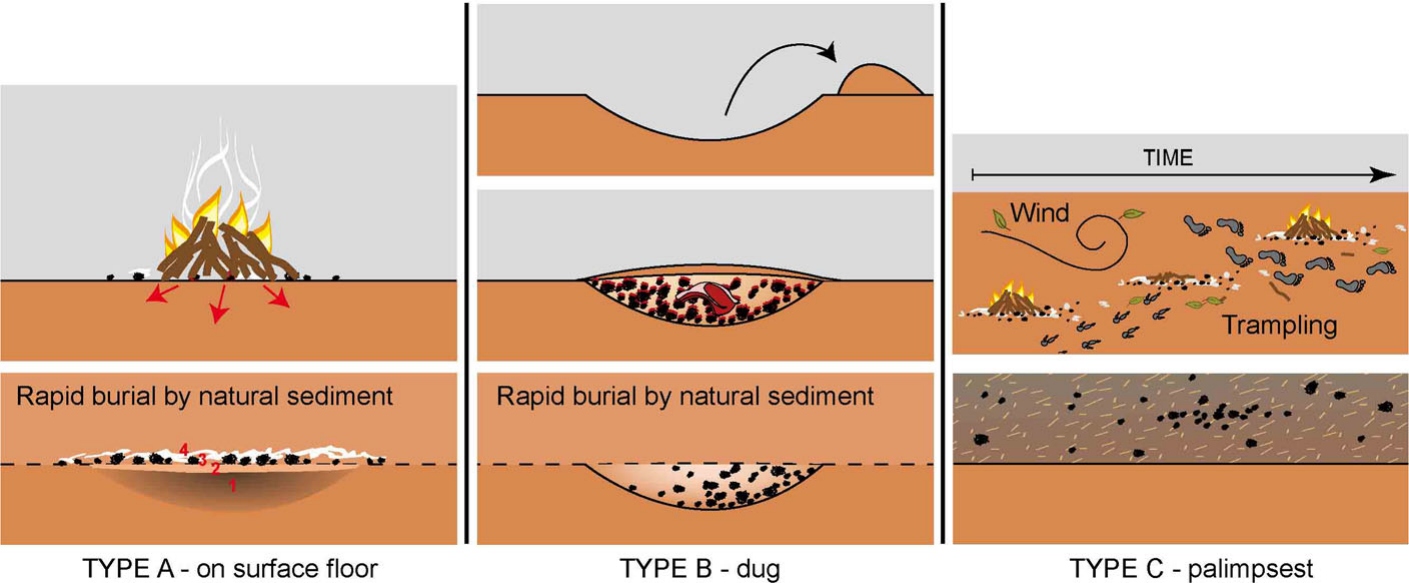
Combustion feature types found in Riwi (modified from Vannieuwenhuyse 2016)

Based on excavation, sediment and anthracological observations, combustion features in the Riwi sequence have been grouped into three different types (Fig. 4; Table 1): flat combustion features (type A), dug combustion features (type B) and palimpsest of combustion features (type C). The features analysed in this study are numbered from top to bottom, with a suffix for the combustion feature type. For example, F1-C is the highest feature in the sequence and a type C combustion feature. The sedimentary and anthracological characteristics of each type of combustion feature are detailed and compared in the following sections. Each type, with the exception of F3-A, appears to be found in subsequent chrono-stratigraphical levels, as demonstrated by the site’s sequence (Figs. 2 and 3).

### Description of Combustion Features in Riwi Sequence

Interdisciplinary analyses conducted on the site have demonstrated that Riwi was occupied on a regular basis from 45 ka up to the European contact period, despite a visibly discontinuous stratigraphic record (Vannieuwenhuyse 2016; Wood *et al.* 2016). The combustion features comprise the main non-material evidence for anthropogenic inputs within the Riwi sequence, and as such, provide exceptional insights into past behaviours over time.

The Pleistocene layers (SU12 to SU5, from the bottom to approximately 20 cm below the surface) have an excellent integrity and reveal the presence of numerous flat combustion features (type A; F8 to F14) appearing at the top of SU12, approximately 40 cm from the bedrock (Figs. 2 and 3). Several dug combustion features (type B; F4 to F7) are visible at the top of the Pleistocene sequence and at the bottom of the Holocene layers. In most of the observed sections, a sharp disconformity places Holocene ash-rich deposit (type C; F1 and F2) directly above the orange Pleistocene layers dated to 34–31 ka. However, discrete episodes of sedimentation have been preserved in some places, in particular, SU3, which includes charcoal and hearths (F3-A) dated to the Last Glacial Maximum (LGM, two charcoal dated at 20620–20040 cal BP, see Table 1) and is only visible in the south-east corner of square 3 (Fig. 2, see also figures in Vannieuwenhuyse 2016; Wood *et al.* 2016). The Holocene layers (F1-C and F2-C) are dated to 7 ka and 0.8–0.7 ka and are mainly composed of an ash-rich accumulation (type C) that encompasses compacted combustion residues (ash, charcoal) and other vegetal organic remains. Preservation of organic material *via* desiccation within the Holocene layers is exceptional and includes seeds, fruit fragments, paperbark fragments (*Melaleuca* spp. bark that had and still has a myriad of uses across Aboriginal groups of Australia, *e.g.* Wynjorrotj *et al.* 2005; Yunupingu *et al.* 1995), wood shavings and two wooden artefact fragments (Dilkes-Hall 2014; Langley *et al.* 2016; Whitau *et al.* 2016b).

### Microstratigraphic Results

**Fig. 5:**
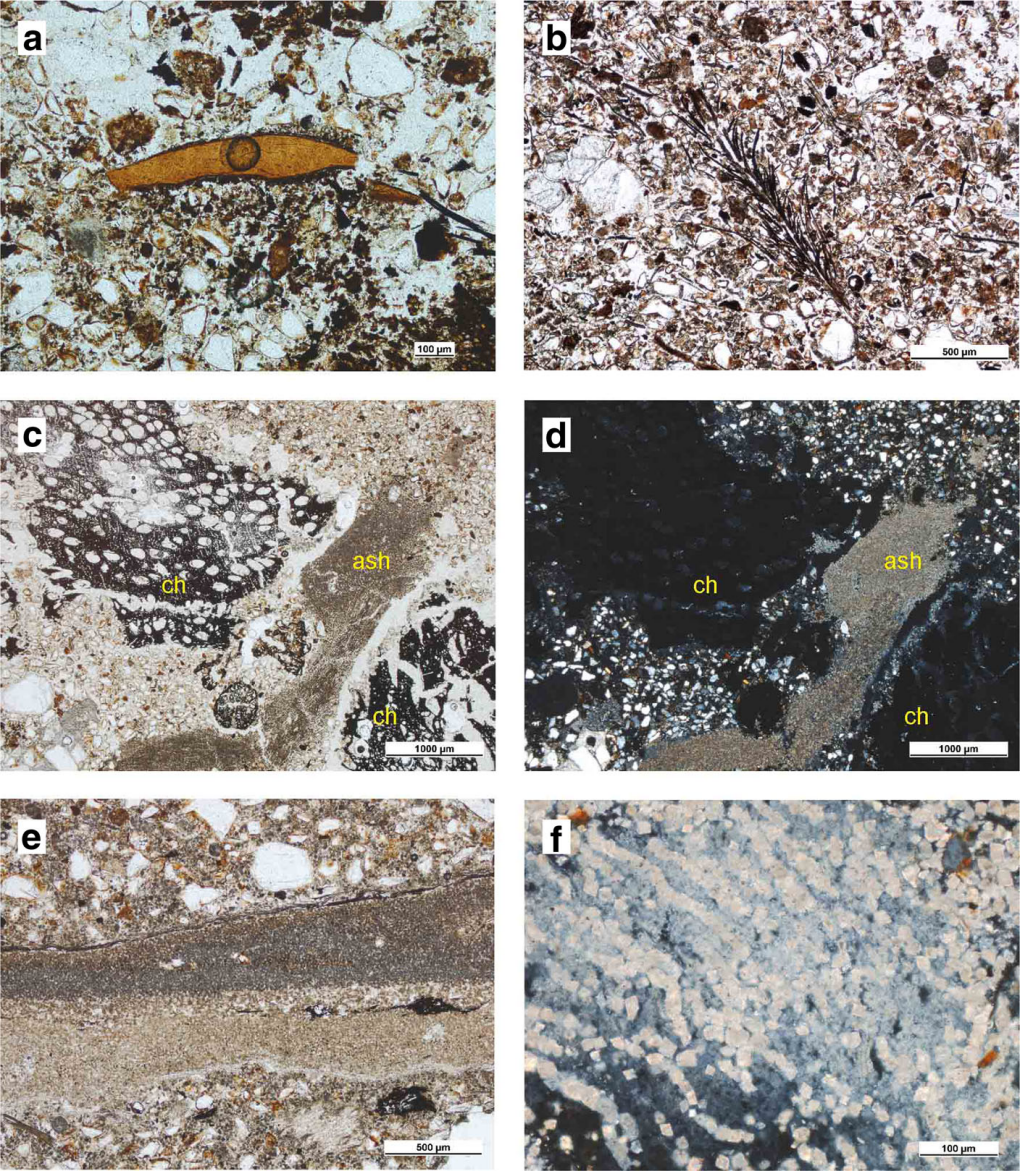
Microphotographs of combustion by-products observed in Riwi thin sections (reproduced from Vannieuwenhuyse 2016). **a** Burnt bone displaying brownish colour (R515B, PPL, scale 100 μm); **b** inflorescence fragment charred by heat of combustion features and microcharcoals mixed in geogenic sediments (F14-A, thin section R502B, PPL, scale 500 μm); **c**, **d** charcoal fragments and ash particles, note the ash calcitic crystal (bright) high interference colours (F6-B, R507C, PPL and XPL, scale 1000 μm); **e** non-disturbed ashes showing colour variation from yellowish to grey and the presence of phytoliths in the outer parts (F2-C, R509A, PPL, scales 500 and 1000 μm); **f** articulated ash particles with typical prismatic shape at high magnification (F2-C, R508B, XPL, scale 100 μm)

The Riwi natural sequence is composed of a mix of geogenic, botanical and animal bone fragments in various proportions (detailed results of the Riwi archaeo-stratigraphical sequence geoarchaeological analysis and a full description of the micromorphological thin sections analysed can be found in Vannieuwenhuyse 2016). While the Pleistocene layers are predominantly composed of geogenic particles, giving the sediment a strong orange hue within which combustion features are easily distinguishable, the Holocene deposit is grey because of the predominant proportion of combusted botanical residues (Figs. 2 and 3). Across the five combustion features sampled for micromorphology (three type A: F11-A, F13-A, F14-A; one type B: F6-B; and one type C: F2-C, see Table 1; Fig. 3), the main combustion by-products observed are vegetal residues (phytoliths, ash, seeds, wood charcoal) and some animal bone fragments (Fig. 5), observed at various burning stages (partially burnt, charred, turned to ash) and in varying states of preservation (depending on syn- and post-depositional modifications, *e.g.* decomposition, alterations).

### Anthracological Results

Across the features sampled (seven type A: F3-A, F8-A, F9-A, F10-A, F11-A, F12-A, F13-A; three type B: F4-B, F5-B, F7-B; and two type C: F1-C, F2-C, see Table 1; Fig. 2), a total of 2824 charcoal fragments were analysed, 1861 of which were positively identified to varying taxonomic ranks. Table 2 lists the positively identified taxa, with their relative frequencies for each feature expressed in terms of absolute fragment counts and the percentages of the total number of fragments analysed. A total of 19 taxa, including two family level identifications (Lamiaceae sp. and Myrtaceae sp.), were determined from nine family groups. A full description of the archaeological charcoal types is presented in Whitau *et al.* (2016a, Appendix 1). Results are discussed in more detail in ‘Types of Combustion Features—Micromorphological and Anthracological Characteristics’.

**Table 2:** Anthracological results from Riwi square 3 for combustion features types A, B and C, expressed in both absolute fragment counts and as percentages of the total number of analysed fragments

Type	Context	Euphorbiaceae	Fabaceae			Lamiaceae		Moraceae			Myrtaceae						Phyllanthaceae	Proteaceae	Simaroubaceae	Ulmaceae	Number of Identifiable Fragments	Indeterminable	Total number of analysed fragments
		*Mallotus* sp.	*Bauhinia* sp.	*Erythrophleum* sp.	*Vachellia* sp.	*Vitex* sp.	Lamiaceae sp.	*Ficus* sp. type A	*Ficus* sp. type B	*Ficus* indeterminate	*Corymbia* sp.	*Eucalyptus* sp. type A	*Eucaltptus* sp. type B	*Eucalyptus* sp. indeterminate	*Melaleuca* sp.	Myrtaceae sp	*Fluegge*a sp.	*Grevillea*/*Hakea* sp.	*Brucea* sp.	*Celtis* sp.			
Absolute fragment counts																							
C	F1-C	45	0	22	0	26	6	13	8	0	163	20	1	5	18	0	34	2	7	3	373	145	518
C	F2-C	6	4	5	6	23	9	23	6	2	163	1	1	1	22	1	27	0	5	23	328	83	411
A	F3-A	1	1	6	2	6	1	2	3	0	44	14	1	0	0	0	3	0	17	0	101	98	199
B	F4-B	3	7	21	3	9	2	7	4	0	138	13	1	3	7	0	22	0	10	10	260	179	439
B	F5-B	1	6	26	7	1	0	0	0	0	156	55	18	11	25	3	2	0	3	3	317	162	479
B	F7-B	0	6	22	2	0	4	1	0	0	163	34	19	20	8	5	4	0	16	5	309	170	479
A	F8-A	0	0	3	0	0	0	0	0	0	2	0	0	0	0	0	1	0	0	0	6	17	23
A	F9-A	0	2	92	28	3	8	0	0	0	12	7	0	2	2	1	0	0	0	1	158	73	231
A	F10-A	0	0	0	0	0	0	0	0	0	0	0	0	0	0	0	0	0	0	0	0	6	6
A	F11-A	0	0	0	0	0	0	0	0	0	0	1	0	1	1	0	0	0	0	0	3	12	15
A	F12-A	0	0	0	0	0	0	0	0	0	0	3	0	0	0	1	0	0	0	0	4	7	11
A	F13-A	0	0	0	0	0	0	0	0	0	1	1	0	0	0	0	0	0	0	0	2	11	13
Proportion of total number of analysed fragments																							
C	F1-C	8.7	0.0	4.2	0.0	5.0	1.2	2.5	1.5	0.0	31.5	3.9	0.2	1.0	3.5	0.0	6.6	0.4	1.4	0.6	72.0	28.0	100
C	F2-C	1.5	1.0	1.2	1.5	5.6	2.2	5.6	1.5	0.5	39.7	0.2	0.2	0.2	5.4	0.2	6.6	0.0	1.2	5.6	79.8	20.2	100
A	F3-A	0.5	0.5	3.0	1.0	3.0	0.5	1.0	1.5	0.0	22.1	7.0	0.5	0.0	0.0	0.0	1.5	0.0	8.5	0.0	50.8	49.2	100
B	F4-B	0.7	1.6	4.8	0.7	2.1	0.5	1.6	0.9	0.0	31.4	3.0	0.2	0.7	1.6	0.0	5.0	0.0	2.3	2.3	59.2	40.8	100
B	F5-B	0.2	1.3	5.4	1.5	0.2	0.0	0.0	0.0	0.0	32.6	11.5	3.8	2.3	5.2	0.6	0.4	0.0	0.6	0.6	66.2	33.8	100
B	F7-B	0.0	1.3	4.6	0.4	0.0	0.8	0.2	0.0	0.0	34.0	7.1	4.0	4.2	1.7	1.0	0.8	0.0	3.3	1.0	64.5	35.5	100
A	F8-A	0.0	0.0	13.0	0.0	0.0	0.0	0.0	0.0	0.0	8.7	0.0	0.0	0.0	0.0	0.0	4.3	0.0	0.0	0.0	26.1	73.9	100
A	F9-A	0.0	0.9	39.8	12.1	1.3	3.5	0.0	0.0	0.0	5.2	3.0	0.0	0.9	0.9	0.4	0.0	0.0	0.0	0.4	68.4	31.6	100
A	F10-A	0.0	0.0	0.0	0.0	0.0	0.0	0.0	0.0	0.0	0.0	0.0	0.0	0.0	0.0	0.0	0.0	0.0	0.0	0.0	0.0	100.0	100
A	F11-A	0.0	0.0	0.0	0.0	0.0	0.0	0.0	0.0	0.0	0.0	6.7	0.0	6.7	6.7	0.0	0.0	0.0	0.0	0.0	20.0	80.0	100
A	F12-A	0.0	0.0	0.0	0.0	0.0	0.0	0.0	0.0	0.0	0.0	27.3	0.0	0.0	0.0	9.1	0.0	0.0	0.0	0.0	36.4	63.6	100
A	F13-A	0.0	0.0	0.0	0.0	0.0	0.0	0.0	0.0	0.0	7.7	7.7	0.0	0.0	0.0	0.0	0.0	0.0	0.0	0.0	15.4	84.6	100

Results for F1-C and F2-C (SU1 and SU2) are reproduced from Whitau *et al.* (2016a)

**Fig. 6:**
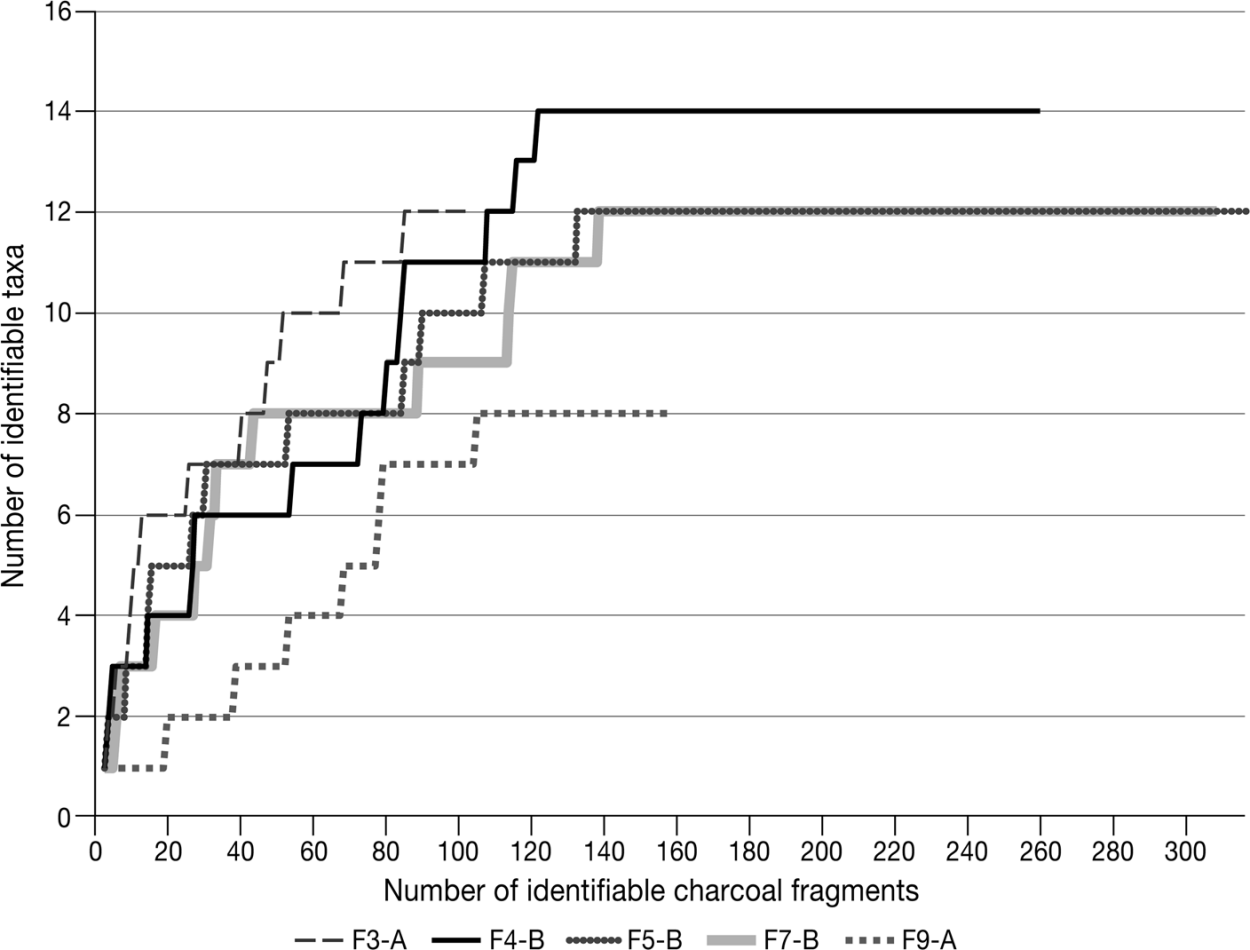
Saturation curves for combustion features types A and B (CAD: CartoGIS, Australian National University)

Saturation curves are presented in Fig. 6, which plot the number of identifiable charcoal fragments against the number of identifiable taxa for each feature. Each plateau indicates the number of fragments that need to be identified in order for the diversity of the archaeological assemblage to be appropriately represented (Byrne *et al.* 2013; Chabal *et al.* 1999; Dotte-Sarout *et al.* 2015; Scheel-Ybert 2002). Across the 12 contexts analysed, seven saturation curves (F1-C, F2-C, F4-B, F5-B, F7-B, F8-A, F9-A) reach plateaux, illustrating that these contexts provide viable representations of the assemblage diversity. The F3-A, F10-A, F11-A, F12-A and F13-A curves do not stabilise. While the F10-A, F11-A, F12-A, F13-A, SU11 and SU12 assemblages comprise a total of 67 identifiable fragments and are excluded from ‘Fuel Wood Management: Comparing Concentrated and Scattered Charcoal Contexts’, the F3-A assemblage, with 101 identifiable fragments, is of a reasonable size, almost reaching the mean plateau point of the other viable sample sizes, and is cautiously included in comparisons with scattered contexts.

### Types of Combustion Features—Micromorphological and Anthracological Characteristics

The following sections describe the sedimentary characteristics and wood charcoal assemblages identified for each of the combustion feature types.

#### Type a Flat Combustion Features (F3-A, F8-A, F9-A, F10-A, F11-A, F12-A, F13-A)

**Fig. 7:**
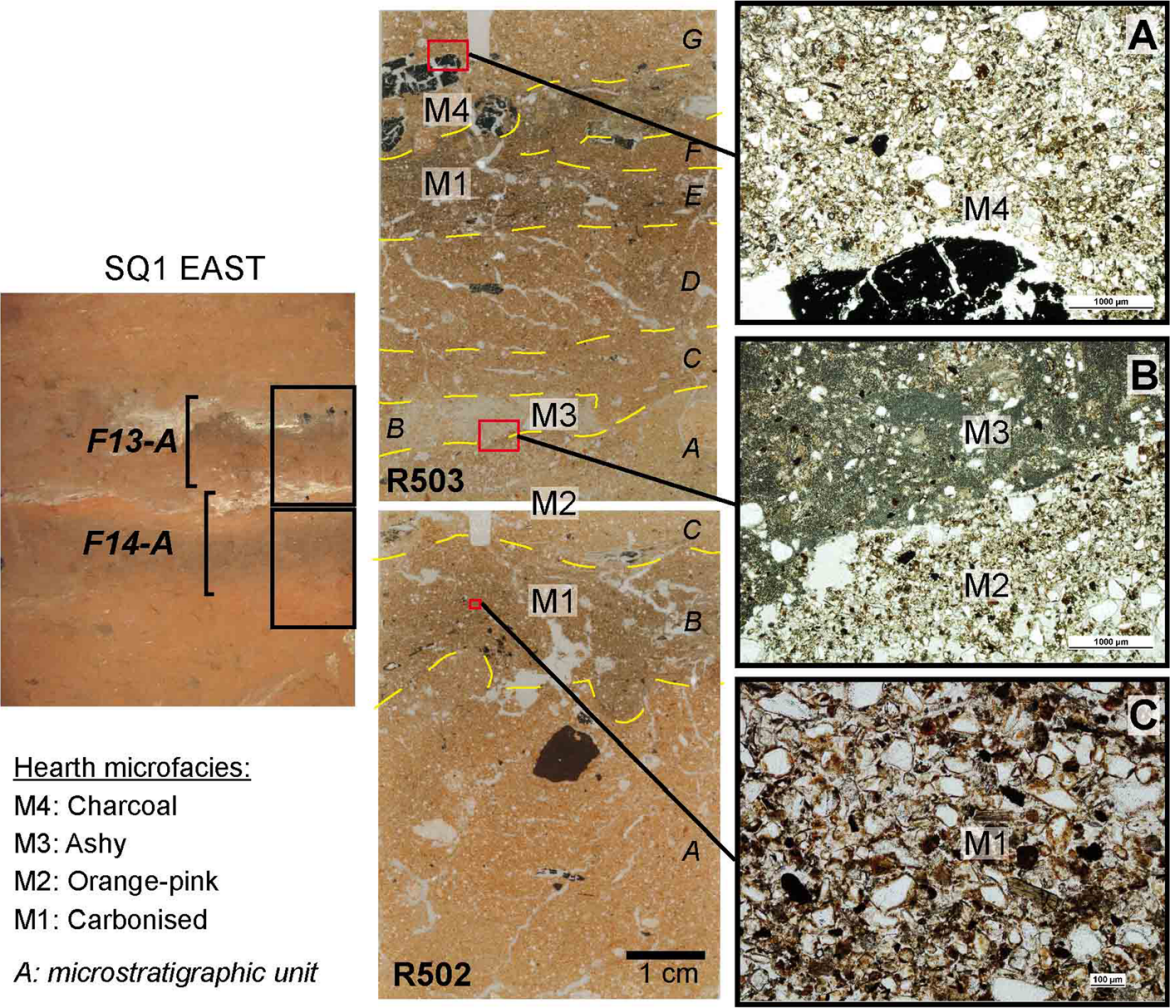
Microfacies types in Pleistocene type A flat combustion features (modified from Vannieuwenhuyse 2016). Left, detailed view of flat combustion features F13-A and F14-A in Riwi square 1 eastern section showing location of micromorphological samples. Middle, scan of thin sections R502 and R503 with microstratigraphic units identified in each thin section, microphotos location and hearth microfacies types (M1 to M4). Right, microphotographs of type A flat combustion features microfacies types (numbering in reference to Fig. 4 and in-text description). **a** Top layer with charcoal fragments (microfacies 3) (F13-A, R503G, PPL, scale 1000 μm); **b** orange-pinkish (microfacies 2) and whitish layers (microfacies 4) (F14-A, R503A/B, PPL, scale 1000 μm); **c** dark carbonised organic-rich layer (microfacies 1) (F14-A, R502B, PPL, scale 100 μm)

Abundant throughout the Pleistocene units dated from 45 to 34 ka (SU11 to SU7), type A combustion features are flat lenses that show a complex layering (Fig. 4). Four different microfacies were identified in type A combustion features (illustrated by micromorphological observations undertaken for F12-A and F13-A, Fig. 7, see also Appendix 1 for micromorphological descriptions of the microstratigraphic units and microfacies), with from bottom to top:

- (1) Dark brown layer (concave shapes visible in stratigraphy) composed of geogenic sands, with a high proportion of microcharcoal and carbonised vegetal particles (Fig. 7c).
- (2) Orange-pinkish layer composed of geogenic sands and ash particles (Fig. 7b).
- (3) Layer with charcoal chunks embedded within the geogenic sands (Fig. 7a).
- (4) Whitish layer (Fig. 7c) mostly composed of ash particles (pseudomorphs of plant calcium oxalate) and phytoliths that are often still in anatomic connection (articulated).

With the exception of F3-A and F9-A, charcoal preservation from each of the analysed type A features is very poor. The few charcoal recovered from F10-A, F11-A, F12-A and F13-A are soft and rounded, with all of the indeterminable fragments unable to be examined due to brittleness, disintegrating to ash particles and providing no clean sections. Of the 45 charcoal recovered from across these four features, only 9 were identifiable, predominantly *Eucalyptus* sp. type A, and all of the Myrtaceae family. By contrast, in terms of both composition and preservation, the F8-A feature produced six identifiable fragments, three were identified *Erythrophleum* sp., two *Corymbia* sp. and one *Flueggea* sp., while eleven of the indeterminable fragments were too brittle and six were too vitrified.

The two type A features which produced reasonable anthracological assemblages are F9-A (*N*_i_ = 158) and F3-A (*N*_i_ = 101). F9-A, which is located at the bottom of SU7 (Fig. 2) is the only Riwi anthracological assemblage that is not dominated by Myrtaceae: *Erythrophleum* sp. (39.8%) dominates with subsidiary *Vachellia* sp. (12.1%), *Corymbia* sp. (5.2%) and *Eucalyptus* sp. (3.8%). F9-A is also anomalous in that it is the only Pleistocene unit to produce an assemblage with one individual taxon count (*Erythrophleum* sp., *N*_i_ = 92) that is higher than the indeterminable count (72 fragments) for that unit. Observed within SU3—a stratigraphic layer dated from the LGM timing (Table 1) that shows evidence of post-depositional bioturbation (Fig. 2)—F3-A is the youngest type A feature analysed and sits above the type B contexts developed in the next section. The dominant taxon for this unit is *Corymbia* sp., with subsidiary *Brucea* sp. and *Eucalyptus* sp. charcoal. Comprising 8.5% of the total charcoal examined within the F3-A unit, *Brucea* sp. charcoal were identified here in their highest proportion of the Riwi anthracological assemblage.

#### Type B Dug Combustion Features (F4-B, F5-B, F6-B, F7-B)

**Fig. 8:**
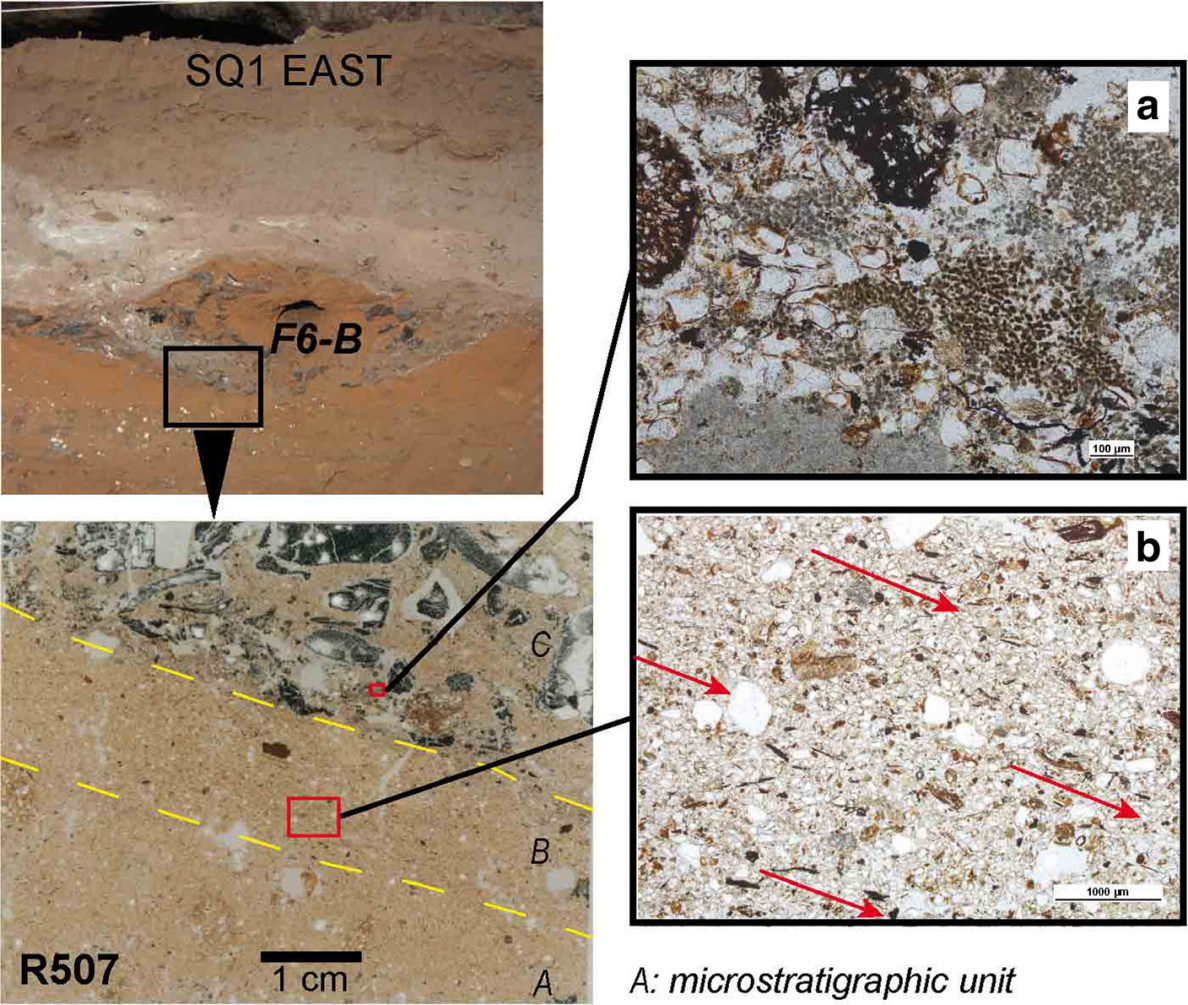
Microfacies types in Pleistocene type B dug combustion features (modified from Vannieuwenhuyse 2016). Top left, detailed view of combustion feature F6-B in Riwi square 1 east section showing location of micromorphological sample. Bottom left, scan of thin section R507 showing the different microstratigraphic units identified. Right, microphotographs of two different microfacies identified. **a** Mixed charcoal fragments, ash particles and geogenic sands (R507C, PPL, scale 100 μm); **b** bedded organic particles below the combustion feature that could indicate digging, the particles being oriented in the same direction due to sloping (R507A, PPL, scale 1000 μm)

Around 34 ka, type B combustion features are observed in section (Figs. 2 and 3). These dug features sometimes cut through the flat combustion features immediately below, disturbing the orientation of particles (Fig. 8b). Type B combustion features contain large fragments of charcoal embedded within a fine matrix of geogenic sands and/or random oriented ash particles (Fig. 8a).

The three type B features selected for wood charcoal analysis (F4-B, F5-B, F7-B) each have a relatively low percentage of indeterminable fragments, ranging from 33.8% for F5-B to 40.8% for F4-B. *Corymbia* sp. is the dominant taxon across the three type B features, with subsidiary *Eucalyptus* sp. and *Erythrophleum* sp. The feature F4-B, which has the lowest proportion of *Eucalyptus* sp. (3.9%) compared with F5-B and F7-B (17.6 and 15.3%, respectively), also has a higher proportion of *Flueggea* sp. (5.0%) than these other type B features, a proportion which is not duplicated in the other Pleistocene contexts.

#### Type C Palimpsest Combustion Features (F1-C, F2-C)

**Fig. 9:**
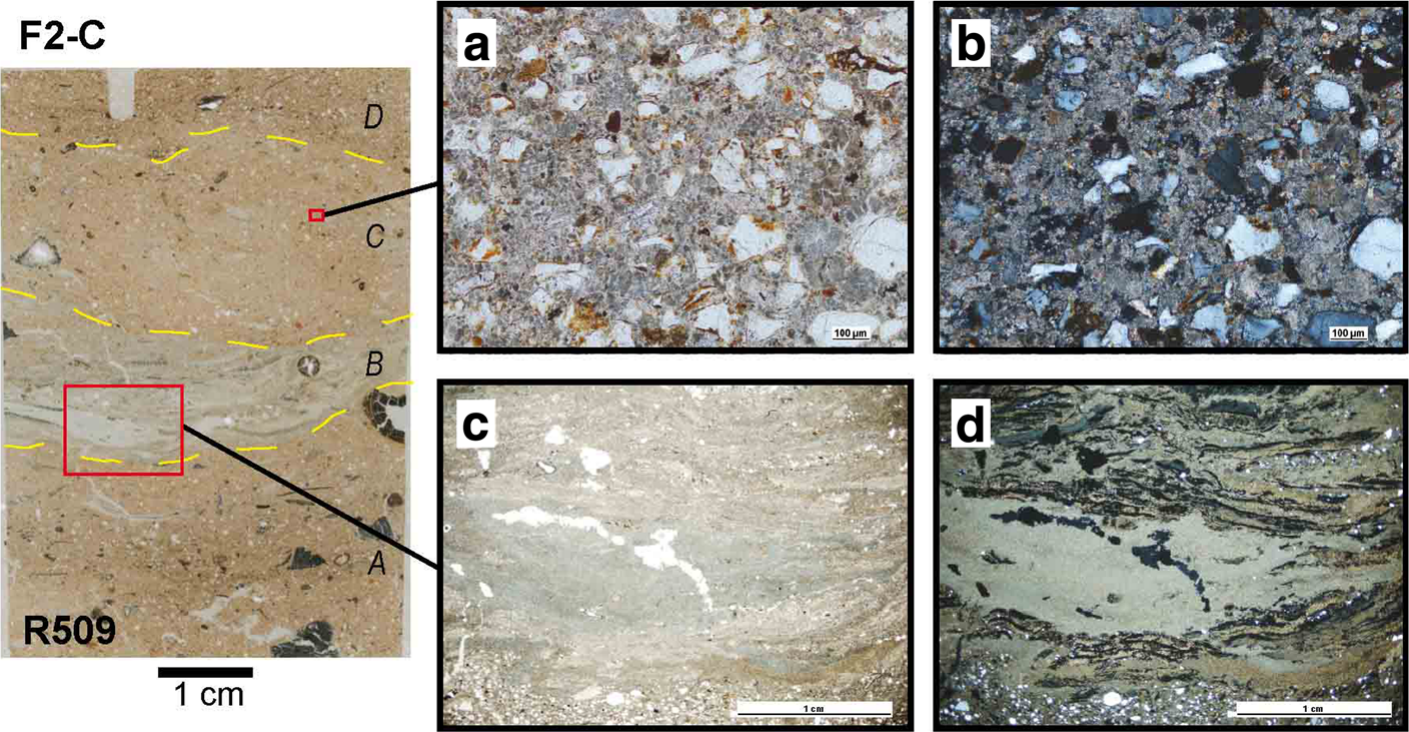
Microfacies types in Holocene type C combustion feature (modified from Vannieuwenhuyse 2016). Left: thin section R509 sampled in SU2 (F2-C) showing microfacies identified. **a**, **b** Mixed facies of non-articulated ash particles and geogenic sands (PPL and XPL, scale 100 μm); **c**, **d** accumulation of non-disturbed ashes including calcitic crystal pseudomorphs of plant calcium oxalate (bright and white in XPL) and isotropic phytoliths (appear dark in XPL because optically isotropic) (PPL and XPL, scale 1 cm)

Type C features are only present in the Holocene deposits and are accumulations composed mainly of by-products of combustion (predominantly ash and charcoal), along with a mix of non-burnt vegetal parts and a minor proportion of geogenic sands (Fig. 9). The high proportion of combustion residues gives the Holocene deposits its grey colour (Figs. 2 and 3). Under a microscope, the microfacies show a very bright calcitic crystallic b-fabric as a result of the high proportion of ash particles (pseudomorphs of plant tissues) (Fig. 9b, d). These particles are found both in anatomic connection (Fig. 9c, d) and disturbed (Fig. 9a, b), which indicates some mixing in this level, resulting from maintenance activities such as reworking (cleaning rake-out of hearths but also human and animal trampling and turbation, see Fig. 4).

Yielding exceptional preservation of organics, Riwi’s two excavated Holocene units, F1-C and F2-C, produced the lowest proportions of indeterminable charcoal of all analysed contexts (28.0 and 20.2%, respectively). Dominated by *Corymbia* sp. (39.7%), with subsidiary *Flueggea* sp. and other dry rainforest taxa, F2-C is the most diverse unit anthracologically (14 taxa). F1-C is broadly similar to F2-C; *Corymbia* sp. (31.5%) dominates with subsidiary *Mallotus* sp. (8.7%) and *Flueggea* sp. (6.6%). The high proportion of *Mallotus* sp. is not duplicated elsewhere in the Riwi anthracological record.

## Discussion: Fire Management at Riwi Cave

### The Building Processes of Combustion Features and Their Hypothetical Functions

#### Type A Flat Combustion Features (F3-A, F8-A, F9-A, F10-A, F11-A, F12-A, F13-A)

The micromorphological evidence demonstrates that the flat combustion features found in the Pleistocene layers were constructed in the same way, where the fire was lit directly onto the ground surface (Fig. 4). The underlying substrate is affected by heat in two ways. The dark colour of the concave dark brown layer (1) is the result of the carbonisation of vegetal organics already present in the geogenic matrix. Their carbonisation was induced by the heat radiance from the fire lit on the ground surface above the sediments. The orange-pinkish layer (2) corresponds to the surface where the fire was lit and where temperatures were high enough to induce both the total combustion of organics already present in the sediment and the typical reddening of the sediment resulting from the oxidation of iron components (Aldeias *et al.* 2016; Canti and Linford 2000). In comparison with layer 1, the lighter hue of layer 2 is also a result of the presence of ash from the layers above.

The top of layers 1 and 2 are the remnants of a ‘past occupation surface’ where the fire was lit (as formulated by Mallol *et al.* 2013b), where people walked and lived in the cave at a certain time in the past. The layer with charcoal (3) corresponds to the combustion itself and contains big charcoal fragments and carbonised organics. Observation of the charcoal sampled for the anthracology analysis shows that they tend to be rounded and brittle in all of the analysed Pleistocene type A flat combustion features (with the exception of the two youngest features F3-A and F9-A). The poor preservation of the charcoal structure is mainly explained by the properties of the wood charcoal itself (differential preservation depending on species, use of wet or dry wood) as already suggested by Théry-Parisot *et al.* (2010). The top whitish layer (4) is composed of ash particles that are mostly still in anatomic connection. The anatomic connection of the ash particles indicates that the firing event was *in situ*, and that these structures were not used repeatedly (Mentzer 2014; Miller *et al.* 2010). The excellent preservation of these structures was permitted by a relatively rapid burial by aeolian natural sedimentation after each firing episode with evidence of only minor post-depositional processes, as demonstrated by the steady sediment deposition rate of Pleistocene levels (Wood *et al.* 2016) and the geoarchaeological analysis of the sequence (Vannieuwenhuyse 2016). Aeolian sedimentation, in conjunction with the post-depositional processes observed in the sequence, in particular the formation of pedogenic gypsum, points to relatively dry conditions over the Pleistocene period. Ash particles were not affected by dissolution, which indicates a relative dryness of the sequence through time (Vannieuwenhuyse 2016).

The fact that only few charcoal fragments are found in most type A combustion features (F8-A, F10-A, F11-A, F12-A, F13-A, see Table 2), and that the combustion by-products associated are mostly ash seems also to indicate that the wood burned in these contexts was mostly completely combusted. As such, this type of combustion feature probably indicates short lived activities or short visits to the cave, where the open air fire was left to burn until the fire died (*cf.* Chabal *et al.* 1999). Two type A features yielded more than 11 identifiable charcoal fragments: F3-A and F9-A (see saturation curves in Fig. 6). If the type A features represent short-lived, episodic combustion events, then these assemblages would be expected to have a lower diversity than their coincident scattered contexts, since the former are hypothesised to represent one or two fuel wood collection trips, the latter many (Asouti and Austin 2005; Byrne *et al.* 2013; Chabal 1990, 1992; Chabal *et al.* 1999; Dotte-Sarout *et al.* 2015; Théry-Parisot *et al.* 2010). The F9-A feature, which yielded 158 fragments of identifiable charcoal, has a low taxa diversity (8 taxa). This low diversity is coupled with the lowest proportion of indeterminable charcoal (31.6%) of any of the Pleistocene units, indicating that this low diversity is not an effect of taphonomic factors acting on the assemblage; supporting the hypothesis that the type A hearth structures are episodic archaeological contexts. The F3-A feature, which sits above the type B features in between the Pleistocene and Holocene sediments, is an exception to this pattern as it yields a relatively high number of identifiable fragments (*N*_i_ = 101) for a type A feature and a diverse assemblage (12 taxa). This feature is discussed in further detail in ‘Fuel Wood Management: Comparing Concentrated and Scattered Charcoal Contexts’ in relation to its context.

Altogether, the micromorphological and anthracological analyses of the type A features indicate that these could have been small open-air hearths typically used as single-firing episodes, for heating, lighting and cooking purposes during short term visits to the site, where the fire was left to burn until the fuel wood was totally combusted. The type A combustion features (excluding LGM F3-A that shows a different pattern and is discussed in further detail in ‘Fuel Wood Management: Comparing Concentrated and Scattered Charcoal Contexts’ in relation to its context) were all rapidly and successively buried by aeolian sedimentation (Vannieuwenhuyse 2016) over a period of 14,000 years (from the first occupation level dated around 45 ka to the top of SU5 around 31 ka), indicating that visitation to the site was episodic and recurrent over many generations.

#### Type B Dug Combustion Features (F4-B, F5-B, F6-B, F7-B)

The type B dug combustion features, which are found in the upper Pleistocene levels (SU7) and date to around 34 ka (Table 1; Wood *et al.* 2016), present completely different sedimentary characteristics to the flat type A combustion features. The type B features contain many more charcoal than the type A flat hearths (Table 1; Fig. 5c, d), indicating a more incomplete combustion process. At the micromorphological scale, the ash particles are generally not observed in anatomic connection, which indicates that the combustion residues have been displaced. Geogenic sand is intimately mixed with the combustion by-products (Fig. 8a) within the structures and could be related to the covering of the fire with sediment (Fig. 4). Baking food in ground ovens is a cooking practice frequently recorded ethnographically amongst some Aboriginal groups (*e.g.* Gould 1968; Harney 1951) and was experienced by one of the authors (RW) during her fieldwork with Gooniyandi traditional owners. The sedimentary facies observed in the structure reflect an earth oven functioning, from the scraping preparation step to the abandonment of the structure in a disrupted state. Indeed, if the type B features were used as cooking pits, which were covered with earth as a ground oven, both the restricted exposure to oxygen and the extinction of the fire at the expected cooking time might explain why combustion of the fuel was incomplete (Antal and Grønli 2003). Such conditions would typically produce larger, more solid charcoal than the type A hearths, which were exposed to oxygen and left to burn until resulting in complete combustion of the wood. Similar dense and dug features have been interpreted as possible cooking pits in Holocene and terminal Pleistocene layers of rockshelter excavations in western and central Australia (Byrne *et al.* 2013; Smith *et al.* 1995), but here, our interpretation is supported by both micromorphological and anthracological lines of evidence.

#### Type C Palimpsest of Combustion Features (F1-C, F2-C)

The Holocene layers are mainly composed of a compact ash-rich deposit, which represents a palimpsest of *in situ* fire places and secondary contexts of ash accumulations (hearth rake-out and ash dumps) in conjunction with material evidence for diverse activities taking place in the cave (botanical remains and artefacts). Often individual features are difficult to distinguish but remnants of types A and B combustion features were both observed throughout these levels. This absence of clear layering is also observed at the microscale, with most of the particles in the Holocene deposits observed in random patterns, with both fresh and carbonised organic matter mixed with geogenic sands and vegetal organic debris, fresh or carbonised (Fig. 9a, b). These observations confirm that intense processes such as trampling, mixing and bioturbation due to the presence of humans and animals in the cave have disturbed the primary organisation of the upper levels of the deposit (Fig. 4).

Based on the microstratigraphic and anthracological analyses as well as archaeological material evidence from the site (Balme 2000; Vannieuwenhuyse 2016; Whitau *et al.* 2016a, b; Wood *et al.* 2016), the Holocene units seem to reflect a more consistent occupation at Riwi than the earlier Pleistocene layers. In other Aboriginal archaeological sites around Australia, the Mid- to Late Holocene is often described as a period of intensification, with various interpretations of changes observed in the archaeological record extrapolated from single site contexts to encompass regional and pan-continental spatio-temporal narratives (see summaries in Langley *et al.* 2011; Ulm 2013). In the Kimberley region, increased abundance in charcoal and lithic artefacts has been argued to signify a more intense occupation during the Holocene (*e.g.* Dortch and Roberts 1996; O’Connor 1995, 1999; Veitch 1996). In terms of lithic artefacts, recent reduction-based analyses (*e.g.* Maloney and O’Connor 2014; Maloney *et al.* 2014) have demonstrated that peaks in lithic artefact discard directly correlate to distinct technological innovations, in line with observations of other assemblages in northern Australia (*e.g.* Clarkson 2008).

### Fuel Wood Management: Comparing Concentrated and Scattered Charcoal Contexts

**Fig. 10:**
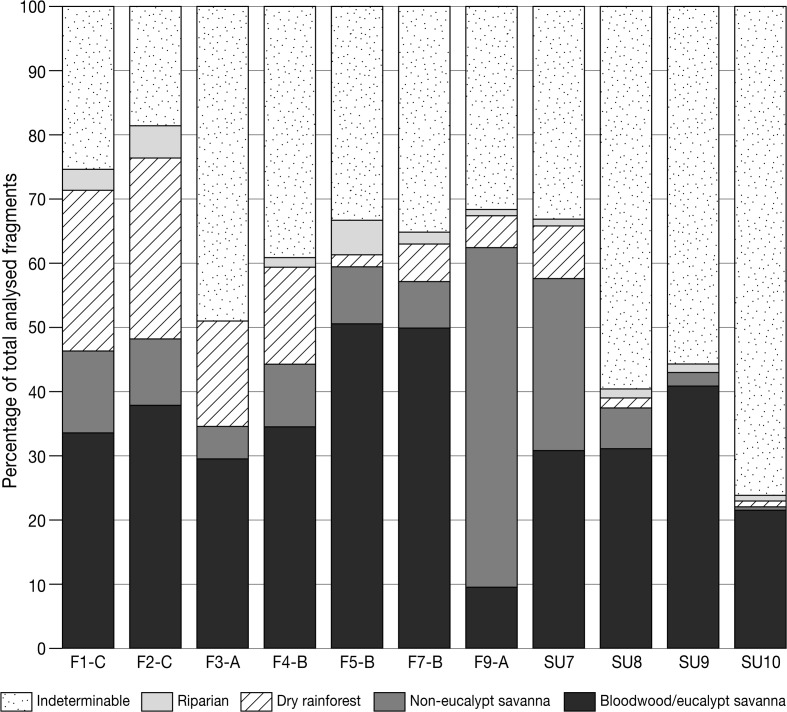
Comparison of wood charcoal assemblage composition between combustion features types A, B and C, and the Riwi scattered contexts presented in Whitau *et al.* (2016a) (CAD: CartoGIS, Australian National University)

**Fig. 11:**
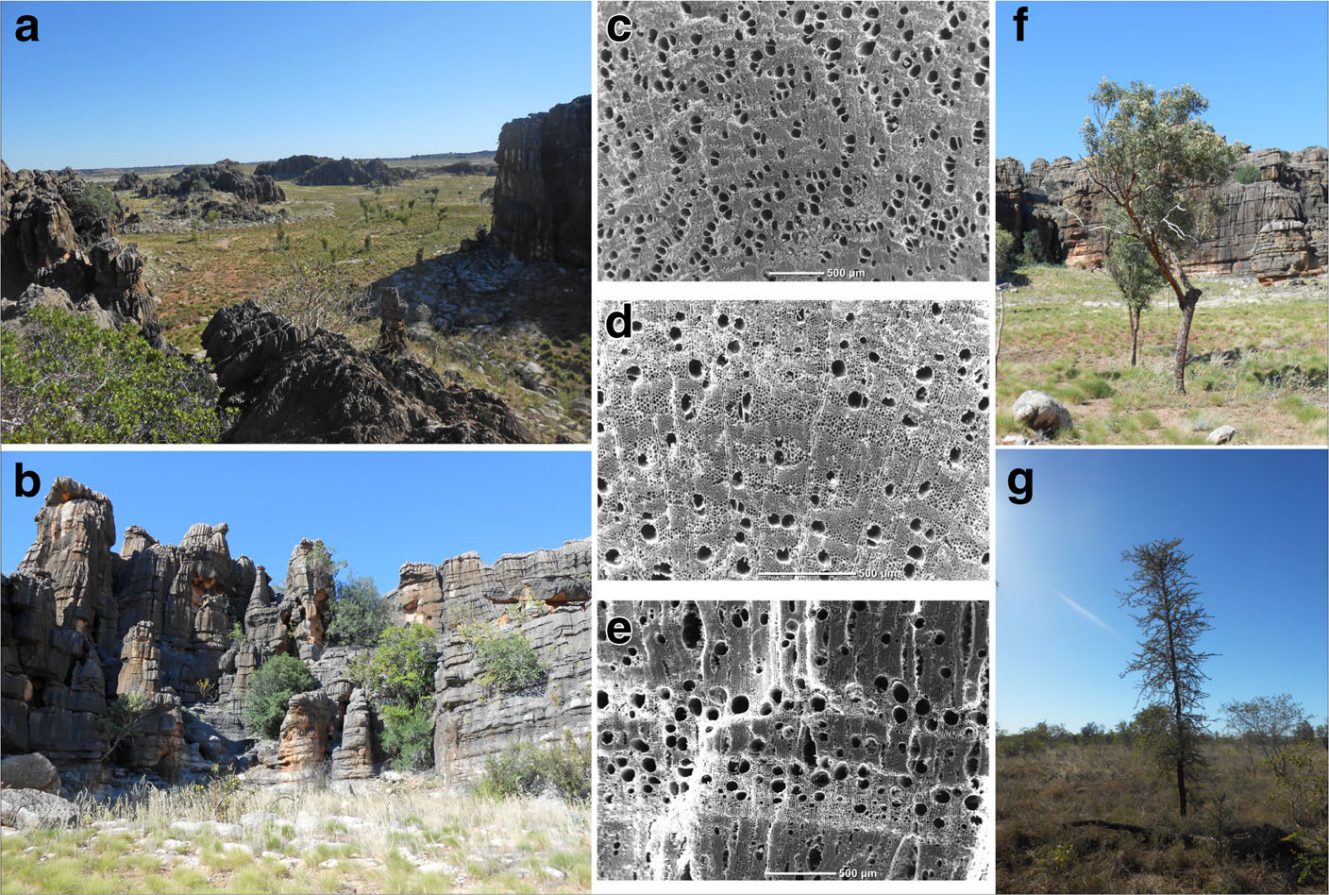
Vegetation types and their common charcoals. Left, low tree steppe of the Riwi valley floor (**a**), dry rainforest taxa colonise the limestone outcrop (b); centre, reference SEM images of *Corymbia dampieri* (**c**), *Vachellia suberosa* (**d**), *Brucea javanica*; right: *Corymbia* sp. (**f**) and *Vachellia suberosa* (**g**) (photos and SEM by R. Whitau)

Table 3 and Fig. 10 present the anthracological results from seven combustion features (F1-C, F2-C, F3-A, F4-B, F5-B, F7-B and F9-A) alongside the stratigraphic contexts SU7, SU8, SU9 and SU10 (reproduced from Whitau *et al.* 2016a) in relation to vegetation type, where taxa have been grouped into five categories: bloodwood/eucalypt savanna, non-eucalypt savanna, dry rainforest, riparian and indeterminable (Fig. 11). Bloodwood/eucalypt savanna is comprised of all the Myrtaceae except for *Melaleuca* sp., which is the sole charcoal type in the riparian category (*e.g.* Fig 11f). Non-eucalypt savanna is composed of those arid-adapted, sclerophyll taxa that colonise the valley floor (*e.g.* Fig. 11g), and the dry rainforest taxa, traditionally associated with monsoonal vine thicket, are those which inhabit the limestone range and outliers (Fig. 11b). The dry rainforest component is comprised of Indo-Malayan taxa, which vary considerably at the family level, in comparison with the deep-time Australian flora (as represented by the other vegetation types) that comprise low familial and high species-level diversity. The dry rainforest taxa are important economically to hunter-gatherer populations (Dilkes-Hall 2014; Whitau *et al.* 2016a).

**Table 3:** Anthracological results expressed in percentages of number of analysed fragments from combustion features F1-C, F2-C, F3-A, F4-B, F5-B, F7-B and F9-A compared with those of scattered contexts SU7, SU8, SU9 and SU10; the latter (and F1-C and F2-C) reproduced from Whitau *et al.* (2016a)

Type	Feature	Bloodwood/eucalypt savanna						Non-eucalypt savannah					Dry rainforest										Riparian	Indeterminable
		*Corymbia* sp.	*Eucalyptus* sp. Type A	*Eucaltptus* sp. Type B	*Eucalyptus* sp. Indeterminate	Myrtaceae sp	**Total bloodwood/eucalypt savanna**	*Bauhinia* sp.	*Erythrophleum* sp.	*Vachellia* sp.	*Grevillea*/*Hakea* sp.	Total non-eucalypt savanna	*Celtis sp.*	*Mallotus sp.*	*Vitex* sp.	Lamiaceae sp.	*Ficus* sp. Type A	*Ficus* sp. Type B	*Ficus* indeterminate	*Fluegge*a sp.	*Brucea* sp.	Total dry rainforest	*Melaleuca* sp.	
C	F1-C	31.5	3.9	0.2	1	0	36.6	0	4.2	0	0.4	13.9	0.6	8.7	5	1.2	2.5	1.5	0	6.6	1.4	27.5	3.5	27.8
C	F2-C	39.7	0.2	0.2	0.2	0.2	40.5	1	1.2	1.5	0	10.8	5.6	1.5	5.6	2.2	5.6	1.5	0.5	6.6	1.2	30.3	5.4	20.1
A	F3-A	22.1	7	0.5	0	0	29.6	0.5	3	1	0	5	0	0.5	3	0.5	1	1.5	0	1.5	8.5	16.5	0	49.4
B	F4-B	31.4	3	0.2	0.7	0	35.3	1.6	4.9	0.7	0	10.2	2.3	0.7	2.1	0.5	1.6	1	0	5	2.3	15.5	1.6	40.4
B	F5-B	32.6	11.5	3.8	2.3	0.6	50.8	1.3	5.4	1.5	0	9	0.6	0.2	0.2	0	0	0	0	0.4	0.6	2	5.2	33.8
B	F7-B	34	7.1	4	4.2	1	50.3	1.3	4.6	0.4	0	7.3	1	0	0	0.8	0.2	0	0	0.8	3.3	6.1	1.7	35.6
A	F9-A	5.2	3	0	0.8	0.4	9.4	0.8	39.8	12.1	0	53.1	0.4	0	1.2	3.5	0	0	0	0	0	5.1	0.8	32
SU	SU7	26.9	3.6	0	1.3	0.4	32.2	0	14.7	8.4	0	28.1	2.1	2.9	0	0.4	1.7	0	0.2	0.4	1	8.7	1.1	34.9
SU	SU8	4.6	12.8	3.1	9.7	0.8	31	0.3	5.6	0.3	0	6.2	0	0	0	0	0	0.3	0	0.3	1	1.6	1.5	59.7
SU	SU9	13.4	16.9	4.3	5.4	0.8	40.8	0	1.9	0	0	1.9	0	0	0	0	0	0	0	0	0.3	0.3	1.3	55.7
SU	SU10	3.2	10.1	2.7	4.3	1.2	21.5	0	0.5	0	0	0.5	0	0	0.3	0.1	0.1	0	0	0.3	0	0.8	0.9	76.3

In Whitau *et al.* (2016a), we argue that firewood was predominantly collected from the valley floor throughout the occupation sequence (Fig. 11a), where the open vegetation would have allowed for easy collection in close proximity to the cave, following the Principle of Least Effort (Shackleton and Prins 1993). Across all features, with the exception of F9-A, firewood was most commonly collected from the bloodwood/eucalypt savanna species of the valley floor (Fig. 11a, f), which was dominated by *Eucalyptus* species, until the time of SU7 deposition, when the vegetation shifted to *Corymbia* sp. dominance, coupled with an increase in shrubby, non-eucalypt savanna taxa (*Erythrophleum* sp. and *Vachellia* sp.) (Fig. 11g) (Whitau *et al.* 2016a). From SU7 onwards, *Corymbia* sp. maintains its dominance in the anthracological record throughout type B and the anomalous F3-A feature until the Early Holocene. The F1-C and F2-C Holocene units reveal that Riwi’s highest charcoal diversity (13 and 14 taxa respectively, see Table 1) is mostly created by larger proportions of dry rainforest taxa and might reflect a different firewood collection strategy, with more fuel collected from the dry rainforest taxa of the limestone outcrops (Fig. 11b). This higher charcoal diversity could also be the result of better preservation of dry rainforest taxa in the upper units of the sequence. The latter seems likely to have been a significant contributing factor, with the exceptional organic preservation of the Holocene units producing the lowest proportions of indeterminable charcoal (F1-C = 28.0% and F2-C = 20.2%, Table 2).

At Riwi, F9-A is the sole context where non-eucalypt savanna taxa dominate the anthracological assemblage, in this case principally represented by *Erythrophleum* sp*.* (Fig. 11g). Even the nine charcoal identified from the F10-A, F11-A, F12-A and F13-A, type A features are all Myrtaceae (Fig. 11c, f) and predominantly *Eucalyptus* sp., which reflects, albeit with very poor assemblage numbers, the composition of the Pleistocene units (SU8–SU10) in which these features were located. The F9-A hearth is positioned at the limit between SU8 and SU7, where a shift in taxa ranks is visible (Fig. 10). SU7 shows an increase in *Corymbia* sp., while *Eucalyptus* sp. types decrease in both abundance and diversity, and *Erythrophleum* sp. and *Vachellia* sp. increase to proportions that are not replicated elsewhere amongst the scattered charcoal assemblages (Whitau *et al.* 2016a). The fuel wood that comprises the F9-A anthracological assemblage was collected before or during the ecological shift observed in the SU7 assemblage, and it is interesting to note that the dominance of *Erythrophleum* in the feature clearly echoes the taxonomic composition shift represented in SU7 (Fig. 10). F9-A is a type A hearth structure, which was not re-used and so the dominance of *Erythrophleum* sp. is most likely an effect of a single firing episode. It could be that *Erythrophleum* sp. was collected in relation to its increased availability in the surrounding landscape where non-eucalypt savanna newly dominated or that it was specifically targeted as a fuel wood, without these hypotheses needing be mutually exclusive.

The *Erythrophleum* genus is composed of some eight (Ross 1998) or nine (Dunlop *et al.* 1995) taxa, one of which, *Erythrophleum chlorostachys* or ironwood, is endemic to Australia, where it grows from northeastern Queensland to the Kimberley region of Western Australia. Aboriginal groups in Queensland and the Northern Territory are recorded using ironwood in a variety of ways: an infusion of the bark is used to treat stomach pains, root infusions for cuts and leaf infusions for scabies; smoke from the wood and leaves is used to relieve constipation and smoke from the bark to produce sterility in women; resin from the roots is an adhesive and gum from the bark is edible and produces a red dye; and the wood is used for carvings, spears, music and cooking sticks (Brock 1988; Dunlop *et al.* 1995; Woinarski *et al.* 2002). While there are no ethnographic accounts of ironwood use in the Kimberley region, at the very least, ironwood is one of the densest of Australia’s native timbers (1200 kg/m^3^) (Boland *et al.* 1984; Woinarski *et al.* 2002) and might provide a different type of fire from the bloodwood/eucalypt species.

The flora represented in the type B features are broadly comparable with those in SU7 in terms of taxonomic diversity, relative frequency and rank of taxa, which is contrary to the expectation that combustion events have low taxonomic diversity, since they represent the last few firing events (Chabal *et al.* 1999; Dotte-Sarout *et al.* 2015; Théry-Parisot *et al.* 2010). The more likely explanation for the similarity between SU7 and the type B dug features (Fig. 10) is that both were used several times and the assemblages represent multiple collecting trips (Whitau *et al.* 2016a). Indeed, F5-B is a series of densely packed combustion features showing concave pits, ash and abundant charcoal; therefore, it securely represents several firing episodes. Such an explanation would also make sense with the proposed identification of these features as ground or earth ovens, with features being re-used several times during an occupation period. Moreover, the voluntary cessation of the combustion process involved in earth oven cooking is an additional factor for a better representation of the original fuel wood taxonomic diversity in comparison with open-air hearth features.

Between the types B and C features sits the F3-A type A hearth. Unlike the other type A contexts, F3-A has a high diversity (12 taxa) in addition to a high proportion of indeterminable charcoal (49.2%) (Table 3). Bloodwood savanna dominates the assemblage, with the high proportion of dry rainforest taxa comprised largely of *Brucea* sp. (8.5%) (Figs. 10 and 11e). The sole species of the genus endemic to Australia, *Brucea javanica* prefers secondary forest, sandy dunes and limestone rock. Generally growing as a small shrub or tree, *B*. *javanica* produces edible roots and fruit that can aid in the treatment of dysentery and fever (Kulip and Wong 1995). Its relatively high abundance in F3-A is not readily explicable and could relate to socio-environmental changes affected by the LGM. The position of the F3-A hearth shows that type A hearth structures continued to be built after the appearance of type B hearths in the record.

While the individual features of the type C units are more difficult to distinguish, both types A and B combustion structures were produced during this time, which is characterised by an increased taxonomic diversity and representation of dry rainforest taxa (Fig. 11b) in the archaeobotanical record, including non-woody remains (Dilkes-Hall 2014; Whitau *et al.* 2016a).

All anthracologically analysed contexts from Riwi cave show that firewood was typically collected from the valley floor and, with the exception of F9-A, bloodwood/eucalypt savanna taxa were the favoured fuels, supporting arguments presented by Whitau *et al.* (2016a). The shift in taxon dominance from eucalypt to bloodwood observed in the scattered contexts, which is likely associated to environmental changes at around 34 ka (Whitau *et al.* 2016a), is reflected in the composition of all the features analysed in this paper.

### Methodological and Archaeological Implications

The study presented here clearly supports important taphonomic studies (*e.g.* Dussol *et al.* 2017; Théry-Parisot *et al.* 2010) in showing that the factors affecting charcoal preservation are complex. Table 4 summarises the anthracological and micromorphological data alongside a summary of the discussion presented above. Fires which were lit directly on the ground surface, like the type A hearth structures, are far less likely to produce quantifiable charcoal within the structure itself, as the direct exposure to oxygen will often combust the fuel entirely. Increase in charcoal abundance in itself should not then necessarily be correlated with an intensification of occupation (Ward *et al.* 2016), as the prevalent type of combustion structure, its formation and length of use as well as its degree of preservation must also be considered. Similarly, changes in deposition dynamics (*e.g.* deficit of natural sedimentation) can lead to the creation of palimpsest type deposits in archaeological sequences (Bailey and Galanidou 2009; Mallol and Mentzer 2015; Vannieuwenhuyse 2016). At Riwi, the changes observed between combustion features, the increased abundance of charcoal dense features within F1-C and F2-C and the high trampling of surface deposits all point towards a change in site use during the deposition of the Holocene units, where occupation of the cave was intensified in terms of the number of site visits, coupled with a reduction of time between visits and lesser accumulation of natural inputs as pointed out in several Australian archaeological studies (*cf.* Ulm 2013; Ward 2004; Vannieuwenhuyse *et al.* 2017).

**Table 4:** Summary of anthracological and micromorphological results

	Type A	Type B	Type C
	Flat combustion features	Dug combustion features	Palimpsest
Combustion feature description	Four different microfacies were identified in type A combustion features (Fig. 7, see Appendix 1.0), with from bottom to top: (1) Dark brown layer (concave shapes visible in stratigraphy) composed of geogenic sands, with a high proportion of microcharcoal and carbonised vegetal particles (Fig. 7c). (2) Orange-pinkish layer composed of geogenic sands and ash particles (Fig. 7b). (3) Layer with charcoal chunks embedded within the geogenic sands (Fig. 7a). (4) Whitish layer (Fig. 7c) mostly composed of ash particles (pseudomorphs of plant calcium oxalate) and phytoliths that are often still in anatomic connection (articulated).	Type B combustion features are characterised by the digging of a pit or depression where the fire was lit and frequently reused. These dug features sometimes cut through the flat combustion features immediately below, disturbing the orientation of particles (Fig. 8b). Type B combustion features contain large fragments of charcoal embedded within a fine matrix of geogenic sands and/or random oriented ash particles (Fig. 8a).	Type C features are accumulations composed mainly of by-products of combustion (predominantly ash and charcoal), along with a mix of non-burnt vegetal parts and a minor proportion of geogenic sands (Fig. 9). The high proportion of combustion residues gives the Holocene deposits its grey colour (Figs. 2 and 3). Under the microscope, the microfacies show a very bright calcitic crystallic b-fabric as a result of the high proportion of ash particles (pseudomorphs of plant tissues) (Fig. 9b, d). These particles are found both in anatomic connection (Fig. 9c, d) and disturbed (Fig. 9a, b) which indicates some mixing in this level.
Hypothesised function	Small, open-air, single-use hearths which were lit directly onto the ground surface and used for heating, lighting, or cooking purposes during short term visits to the site.	Earth or ground ovens which were constructed by digging a pit or depression into the substrate. A fire was lit within the depression and subsequently buried during a hypothesised underground cooking process.	A palimpsest of numerous type A and B features coupled with an increase of combustion residues resulting from maintenance activities such as reworking (cleaning rake-out of hearths but also human and animal trampling and turbation, see Fig. 4).
Economy of wood	The charcoal within the type A combustion features is mostly completely combusted with the exception of the F3-A and F9-A assemblages. F9-A is the only anthracological sample from Riwi Cave which was not dominated by bloodwood/eucalypt; the dominance of *Erythrophleum* sp., coupled with the low diversity of F9-A taxa, is indicative of a single firing episode. Taxa were collected predominantly from the valley floor as per the associated scattered Pleistocene contexts.	The taxonomic diversity of the type B dug features is similar to the associated scattered contexts and might indicate re-use of the combustion features. The dominance of bloodwood savanna taxa indicates that firewood was collected predominantly from the valley floor, with a minor presence of dry rainforest taxa, which are associated with the limestone outcrop.	The savanna taxa of the valley floor continue to be well represented alongside a higher proportion of dry rainforest taxa, which indicates a potentially more extensive use of the surrounding landscape and its various ecological niches.
Inference of hunter gatherer strategies	Occupation was intermittent from 47 to 34 ka, with enough time between site visits to allow for these flat, open-air, single-use hearths to be covered by natural, rapid aeolian deposition.	More frequent and/or longer occupation at the site during this time (c. 34 ka).	Frequent occupation of longer duration during the Holocene sequence producing a palimpsest of archaeological deposition, which forms this type C secondary context.

This study also signifies the importance of understanding site formation processes; as already stated by several Australian researchers (Holdaway *et al.* 2008; Langley *et al.* 2011; Vannieuwenhuyse *et al.* 2017; Ward and Larcombe 2003; Ward *et al.* 2016). The exceptional preservation of flat type A hearths in Riwi provide an archaeological case study for how heating can impact underlying sediments in this type of fine sand substrate, in the Riwi example: several centimetres below the surface where the fire was lit. This depth of effect has several implications in how to sample such features and how to interpret the archaeological material (botanical remains, bones, lithic artefacts, ochre pigments, but also sediment samples for luminescence dating) from the affected underlying substrate, as already pointed out by microstratigraphic experimental studies by Aldeias *et al.* (2016) and Mallol *et al.* (2013b).

## Conclusions

Starting 45 ka years ago and for over 14,000 years, Pleistocene occupation at Riwi was intermittent and potentially episodic. The cave would be visited for short periods: a fire lit directly on the ground (type A), fuelled by wood collected from the eucalypt savanna of the valley floor, and abandoned, with these hearth structures most likely not re-used. Occupation was intermittent, with enough time between site visits to allow for these flat, open-air, single use hearths to be covered by natural, rapid aeolian deposition.

From around 34 ka to the onset of the LGM, a new combustion feature (type B) is observed, which potentially reflects a different type of occupation at the site. Instead of lighting the fire directly on the ground, the fire was lit in a dug pit, covered with earth during combustion and extinguished prior to the completion of the combustion process; in a pattern similar to a ground oven. The anthracological composition of these type B structures is most similar to the scattered context of SU7, demonstrating the collection of fuel wood from the bloodwood-dominated savanna newly established on the valley floor. The taxonomic diversity of the type B dug features might illustrate that these ground ovens were re-used multiple times and potentially signify more frequent and/or longer occupation at the site during this time.

Type A and B combustion features continued to be produced during the LGM and through to the Holocene, when the site seems to have been visited more frequently. Change in deposition patterns and higher frequency of occupation produced a palimpsest of archaeological deposition, which forms the type C secondary contexts. This period also records an increase in use of vegetation resources located in the dry rainforest areas. The savanna taxa of the valley floor continue to be well represented, indicating a potentially more extensive use of the surrounding landscape in its various ecological niches. This aligns with an intensification of occupation, where intensification refers to an increase in the number of site visits and the duration of these visits.

Combined anthracological and micromorphological analyses of combustion features at Riwi have allowed us to propose a typology of features, to define the chronology of their appearance in the record and to document changes in site occupation patterns and landscape use over time. Our results show that interpretations of anthracological spectra should be adapted to the type of combustion structure recovered; the relationship between charcoal preservation and context is far too complex to warrant the direct association of charcoal abundance with intensification of site use and/or population increase.

Future experimental studies which explore both hearth building processes and fuel wood selection strategies using traditional Aboriginal methods will enable a deeper understanding of how fire was manipulated in the past and strengthen the functional interpretation of the different types of combustion features identified in our study. Precise and multiproxy studies allow a few tangible glimpses into the lives of a site’s past inhabitants, building a home ‘where the hearth is’.

## Electronic Supplementary Material

ESM 1 (DOC 162 kb)
