# Supplementary material for: Home Is Where the Hearth Is: Anthracological and Microstratigraphic Analyses of Pleistocene and Holocene Combustion Features, Riwi Cave (Kimberley, Western Australia)
Source: J Archaeol Method Theory. 2017 Oct 26;25(3):739–76. doi: 10.1007/s10816-017-9354-y (PMC6061027; doi:10.1007/s10816-017-9354-y)
Supplement: Supplementary file 1 — (DOC 162 kb) [file 10816_2017_9354_MOESM1_ESM.doc]

| **SU and transition between SU** | **Thin section** | **Microstratigraphic unit** | **Common characteristics**:  Compact complex single-space grain microstructure composed mostly of pellicular grains (with complete or partial inherited clay coatings) and intergrain micro-aggregates. Weak porosity (close packing, 10%). Mineral components composed of mainly sand-sized grains, dominated by quartz, but minor presence of some feldspars, elongated micas (biotite and muscovite) and calcareous sand-sized grains. Clay is mainly present in clay coatings around quartz grains. Bimodal sorting, with a majority of aeolian subangular well-sorted very fine sand, and some rounded medium and coarse sands (from weathered sandstone). Average particle size distribution (fraction under 2mm estimated in thin section and by sieving): Gravel: 0-2%, CS: 0-2%, MS: 2-5%, FS: 2-10%, VFS: 20-50%, Silt: 20-40%, Clay: 5-10%.  Granostriated and undifferentiated birefringence fabric, changing to calcitic crystallic in microfacies containing high proportion of ash. Complex organo-mineral in intergrain micro-aggregates (clay and amorphous organic matter).  Variable presence of phytoliths, tissue residues and organic matter, macro-charcoal fragments and microcharcoal, calcitic ash (generally square or rhomboedric shape, size ~20 µm), microbones (>2 mm), phosphatic aggregates (animal droppings), bird eggshell, malacofauna, insect mud channels.  Secondary carbonates and gypsum nodules in various proportions in the different levels. |
| --- | --- | --- | --- |
| **2** | 509 | D | Yellow-grey ashy mix of natural and anthropogenic sediments characterised by a high proportion of ash (50-80%) giving the sediment a calcitic crystallic birefringence fabric. Some big charcoal fragments present in 508B and 509A (different thin section but same microfacies), 509B is composed of non-disturbed combustion residues, with more than 80% of articulated ash particles. |
|  | 509 | C |
|  | 509 | B |
|  | 509 | A |
|  | 508 | B |
|  |  |  |
| **2/6** | 508 | B | Transition between SU2 and SU6 is clear and mainly identified by the change in proportion of charcoal fragments and ash between 508A and 508B: less packed CH and higher proportion of ash particles are observed in 508B. |
|  | 508 | A |
| **6** | 508 | A | Packed geogenic orange sands with high proportion of large charcoal fragments (1-2 cm) and medium proportion of ash particles. |
|  | 507 | C |
| **6/7** | 507 | B | The boundary between SU6 and SU7 is very sharp at a macroscale level of observation and also very sharp under the microscope between 507B and 507C but there is a transitional microfacies 507B showing elongated bedded organic matter following the feature outline and some ash particles from upper microfacies 507C (SU6). |
| **7** | 507 | A | Packed geogenic orange sands with weak proportion of tissue residues, medium proportion of charcoal, some ash related to combustion features, few microbones, some phosphatic nodules, possible presence of gypsum. |
|  |  |  |
|  |  |  |
| **11** | 504 | A>E | Packed geogenic orange sands with some gypsum nodules (size generally from <1 mm to 3 mm). Low proportion of tissue residues, low proportion of charcoal, some ash particles associated with combustion features, few microbones, some phosphatic nodules. The gypsum nodules are composed of bigger gypsum crystals (>20 µm up to 200 µm, crystal size generally increasing with depth).  502A, 502B, 502C, 503A and 503B form a combustion feature. 503D to 503G form another combustion feature, both features are interspersed within the natural sedimentation.  Passage features were observed in some microfacies of the lower half of SU11 (503F, 502C, 502B).  The transitional microfacies (502B) with SU 12 is marked by a high proportion of microcharcoal, charcoal fragments and charred particles. |
|  | 503 | A> G |
|  | 502 | C |
|  | 502 | B |
|  |  |  |
|  |  |  |
|  |  |  |
|  |  |  |
|  |  |  |
|  |  |  |
| **11/12** | 502 | A | Some passage features were observed at the transition between 502A and 502B, indicating some bioturbation. |
|  |  |  |

Table X1. Riwi micromorphological description of microstratigraphic units identified in the thin sections analysed for this study. Results are presented by major stratigraphic unit SU (data from Vannieuwenhuyse, 2016). See Table X2 for detailed semi-quantitative data for each microstratigraphic units.

Table X2. Riwi micromorphological observations by microstratigraphic units from the thin sections analysed for the study (data from Vannieuwenhuyse, 2016).

|  | |  |  |  |  | Structure | | | Micromass |  | Mineral (%) Visual estimation | | | | | | | |  | Vegetal | | | | | | | Animal | | | | | Post-depositional processes | | | | | | | | | | |
| --- | --- | --- | --- | --- | --- | --- | --- | --- | --- | --- | --- | --- | --- | --- | --- | --- | --- | --- | --- | --- | --- | --- | --- | --- | --- | --- | --- | --- | --- | --- | --- | --- | --- | --- | --- | --- | --- | --- | --- | --- | --- | --- |
| SU | | Wall | Thin section | Microfacies | Lower transtiion | Microstructure | Coarse/fine distribution | Porosity (voids %) | Colour under PPL | Birefringence | Gravel >2mm | Coarse Sand 500µm-2mm | | Medium Sand 250-500µm | Fine Sand 125-250µm | Very Fine Sand 63-125µm | Silt 4-63µm | Clay | (Roundness (Qz) | Plant Residue | Phytoliths | CH<500µm | big CH>500 µm | Ashes |  | | Bones | Phosphatic nodules | Malacofauna | Avian Eggshell | Burning/Heating | Bioturbation | CaCO3 | Gypsum aggregate size Big > 3mm | Medium 1-3 mm | Small <1 mm | Gypsum crystals size Fine <20µm | Medium 20-100µm | Large>50µm | Desert rose like > 200µm | | Bassanite? |
| 2 | | E | 509 | D | C | Compact complex grain | Single-spaced partial chitonic | Close packing (10% voids) | orange-grey | Cc | 0-5 | 0-2 | | 2-5 | 2-10 | 20-50 | 20-40 | 5-10 | ALL SA, (R) | 5 | 1 | 3 |  | 4 | M | | 3 |  |  |  |  |  |  |  |  |  |  |  |  |  | |  |
|  |  | E | 509 | C | S |  |  |  | yellow-grey | Cc | 0 | <2 | | 2 | 2 | 20 | 20-30 | 50 |  | 1 | 2 | 3 |  | 6 | M | | 1 |  |  | ● |  |  |  |  |  |  |  |  |  |  | |  |
|  |  | E | 509 | B | S |  |  |  | pale yellow-grey | Cc | 0 | 0 | | 0 | 0 | 2 | 2-5 | 0 |  | 2 | 5 | 1 |  | 7 | C | |  |  |  |  |  |  |  |  |  |  |  |  |  |  | |  |
|  |  | E | 509 | A |  |  |  |  | yellow-grey | Cc | <2 | <2 | | 2 | 2 | 20 | 20 | 50 |  | 1 | 2 | 5 | 2 | 6 | M,C | | 1 |  |  |  |  |  |  |  |  |  |  |  |  |  | |  |
|  |  | E | 508 | B | C |  |  |  | yellow-grey | Cc | <2 | <2 | | 2 | 2 | 20 | 20 | 10 |  | 1 | 2 | 5 | 3 | 6 | M,C | | 2 |  |  |  |  |  |  |  |  |  |  |  |  |  | |  |
| 6 | | E | 508 | A |  |  |  |  | Orange | Gs, U | <2 | 0 | | 2 | 2 | 40 | 40 | 15 |  | 1 | 2 | 5 | 5 | 3 | M,C | |  |  |  |  |  |  |  |  |  |  |  |  |  |  | |  |
|  |  | E | 507 | C | S |  |  |  | Orange |  | <2 | 2 | | 2 | 2 | 40 | 40 | 15 |  | 1 | 2 | 5 | 5 | 3 | M,C | | 1 |  |  |  |  |  |  |  |  |  |  |  |  |  | |  |
| 7 | | E | 507 | B | C |  |  |  | Orange |  | <2 | 0 | | 2 | 2 | 40 | 40 | 15 |  | 1 | 2 | 3 |  | 2 | M | | 1 |  |  |  |  |  |  |  |  |  |  |  |  |  | |  |
|  |  | E | 507 | A |  |  |  |  | Orange |  | <2 | 0 | | 2 | 2 | 40 | 40 | 15 |  | 1 | 2 | 3 |  |  |  | | 1 | 1 |  |  |  | ● |  |  |  |  |  |  |  |  | | ? |
| 11 | | E | 504 | E | D |  |  |  | Orange | Gs, U | 0 | 0 | | 2 | 2 | 20 | 50 | 15 |  | 1 | 2 | 2 | 1 |  |  | | 1 | 1 |  |  |  |  |  |  | ● | ● |  | ● |  |  | |  |
|  |  | E | 504 | D | D |  |  |  | Orange | Gs, U | 2 | 0 | | 2 | 2 | 20 | 50 | 15 |  | 1 | 2 | 2 |  | 2 | M | | 1 | 1 |  |  |  |  |  |  | ● |  |  | ● |  |  | | ● |
|  |  | E | 504 | C | D |  |  |  | Orange | Gs, U | 0 | 0 | | 2 | 2 | 20 | 50 | 15 |  | 1 | 2 | 1 |  |  |  | | 1 | 1 |  |  |  |  |  |  | ● |  |  |  | ● |  | |  |
|  |  | E | 504 | B | D |  |  |  | orange-brown | Gs, U | 0 | 0 | | 2 | 2 | 10 | 50 | 15 |  | 1 | 2 | 2 |  |  |  | | 2 | 1 |  |  |  |  |  |  |  |  |  |  | ● |  | | ? |
|  |  | E | 504 | A | D |  |  |  | Orange | Gs, U | 0 | 0 | | 2 | 2 | 10 | 50 | 15 |  | 1 | 2 | 1 |  |  |  | | 1 | 1 |  |  |  |  | ? |  | ● | ● |  |  | ● | ● | | ● |
|  |  | E | 503 | G | C |  |  |  | Orange | Gs, U | 0 | <2 | | 2(-5) | 2-5 | 20 | 50 | 20 |  | 1 | 2 | 3 | 2 | 2 | M,C | | 1 |  |  |  |  |  |  |  |  | ● |  |  | ● | ● | |  |
|  |  | E | 503 | F | D |  |  |  | orange-brown | Gs, U | 0 | 0 | | 2 | 2 | 10-20 | 50 | 20 |  | 1 | 3 |  |  |  |  | |  |  |  |  |  | ● |  |  |  |  |  |  |  |  | |  |
|  |  | E | 503 | E | D |  |  |  | Brown | Gs, U | <2 | 0 | | 2 | 2-5 | 20 | 50 | 20 |  | 1 | 4 | 2 |  |  |  | |  |  |  |  |  |  |  |  | ● |  |  |  | ● |  | |  |
|  |  | E | 503 | D | D |  |  |  | Orange | Gs, U | <2 | 0 | | 2 | 2-5 | 20 | 50 | 20 |  | 1 | 2 | 2 |  |  |  | | 1 |  |  |  |  |  |  |  | ● |  |  |  | ● |  | |  |
|  |  | E | 503 | C | S |  |  |  | orange-grey | Cc | 0 | 0 | | 2 | 5 | 10 | 5 | 2 |  | 1 | 2 | 1 |  | 3 | M | | 1 |  |  |  |  | ● |  |  | ● |  |  |  | ● |  | |  |
|  |  | E | 503 | B | C |  |  |  | pale yellow-grey | Cc | 0 | 0 | | 2 | 5 | 10 | 5 | 2 |  | 1 | 2 | 1 |  | 6 | C,M | | 1 | 1 |  |  |  |  |  |  |  | ● |  | ● |  |  | |  |
|  |  | E | 503 | A |  |  |  |  | orange pale | Cc | 0 | 0 | | 2 | 2-5 | 20 | 50 | 20 |  | 1 | 2 |  |  | 2 | M | | 2 |  |  |  |  |  |  |  |  |  |  |  |  |  | |  |
|  |  | E | 502 | C | D |  |  |  | orange pale | Cc | 0 | 2 | | 5-10 | 2 | 20 | 60 | 5 |  | 1 | 2 |  |  | 2 | C,M | | 1 |  |  |  |  |  |  |  |  |  |  |  |  |  | |  |
|  |  | E | 502 | B | C |  |  |  | brown | Gs, U | 0 | 2 | | 2 | 2 | 30 | 50 | 5 |  | 1 | 2 | 2 |  | 3 | M,C | | 1 | 1 |  |  |  | ● |  | ● | ● | ● |  |  | ● | ● | |  |
| 12 | | E | 502 | A |  |  |  |  | orange | Gs, U | 2 | 2 | | 5 | 2 | 30 | 50 | 5 |  | 1 | 2 |  |  |  |  | | 1 | 1 |  |  |  |  |  | ● | ● |  |  |  | ● | ● | |  |
|  | SU= Stratigraphic Unit  PPL=Plain Polarized Light (plain light)  Wall: E= East, S= South, W= West, N= North  Lower transition  D= diffuse  C= clear  S= sharp  Porosity  CP= close packing  Coarse/fine distribution  SSFE= Single-spaced fine enaulic  DSFE= Single-spaced fine enaulic  OFE= Open spaced fine enaulic  C= Chitonic | | | | | | | | | | | | Birefringence fabric  U= Undifferentiated  Gs= Granostriated  Cc= Crystallic calcitic,  Cg= Crystallic gypsum;  Roundness Quartz grains  A= angular  SA= subangular  SR= subrounded  R= rounded  Sorting grains  VW= very well-sorted  W= well-sorted  M= moderately well-sorted  P= poorly sorted | | | | | | | | | | | | | Components occurrence  ?= probable / identification unsure.  ●= present / single occurrence  1= rare <2%  2= occasional 2-5%  3= many 5-10%  4= abundant 10-20%  5= very abundant >20%  6= dominant >50%  7= very dominant=>80%  Ashes: M= mixed, C= in anatomical connexion  Post-depositional processes  ?= probable / identification unsure  ● = present, weakly developed  ●●= developed  ●●●= strongly developed | | | | | | | | | | | | | | |  | |
